# Joint modelling of wheeze and lung function from childhood to early adulthood: four population-based birth cohorts

**DOI:** 10.1016/j.eclinm.2026.103996

**Published:** 2026-05-29

**Authors:** Anhar Ullah, Sara Fontanella, Raquel Granell, Lesley Lowe, Hasan Arshad, Clare S. Murray, Steve Turner, John W. Holloway, Gang Wang, Angela Simpson, Graham Roberts, Erik Melén, Adnan Custovic

**Affiliations:** a National Heart and Lung Institute, Imperial College London, UK; b NIHR Imperial Biomedical Research Centre, London, UK; c Department of Population Health Sciences, Bristol Medical School, University of Bristol, UK; d Division of Infection, Immunity and Respiratory Medicine, School of Biological Sciences, Faculty of Biology, Medicine and Health, University of Manchester, Manchester Academic Health Science Centre, UK; e Human Development and Health, Faculty of Medicine, University of Southampton, Southampton, UK; f NIHR Southampton Biomedical Research Centre, University Hospitals Southampton NHS Foundation Trust, Southampton, UK; g David Hide Asthma and Allergy Research Centre, Isle of Wight, UK; h Royal Aberdeen Children's Hospital NHS Grampian Aberdeen, AB25 2ZG, UK; i Child Health, University of Aberdeen, Aberdeen, UK; j Department of Integrated Traditional Chinese and Western Medicine, West China Hospital, Sichuan University, Sichuan, China; k Department of Clinical Science and Education, Karolinska Institutet, Södersjukhuset, Stockholm, Sweden; l Sachs' Children and Youth Hospital, Södersjukhuset, Stockholm, Sweden

**Keywords:** Lung function, Growth phase, Wheeze phenotypes, Reduced lung function, Birth cohorts, Trajectories, Joint modelling, ALSPAC

## Abstract

**Background:**

Wheeze and lung function (LF) during childhood are key indicators of respiratory health, yet their trajectories are usually examined separately. We aimed to identify joint developmental patterns of wheeze and LF.

**Methods:**

We used data from four unselected birth cohorts established between 1989 and 1996 with repeated assessments of wheeze from infancy and spirometry from early school-age to early adulthood. We used group-based multi-trajectory modelling to derive trajectories based on joint modelling of current wheeze and forced expiratory volume in 1 s/forced vital capacity ratio (FEV_1_/FVC).

**Findings:**

In the discovery analysis (n = 4645), we identified 6 trajectories: (1) Never/infrequent wheeze with normal LF (NIFW-NLF, 2925/4645 [62.97%]); (2) Never/infrequent wheeze with reduced LF (NIFW-RLF, 475/4645 [10.22%]); (3) Early-transient wheeze with normal LF (ETW-NLF, 559/4645 [12.03%]); (4) Late-onset wheeze with NLF (LOW-NLF, 335/4645 [7.21%]); (5) Persistent wheeze with NLF (PEW-NLF, 202/4645 [4.34%]); and (6) PEW with RLF (PEW-RLF, 149/4645 [3.21%]). Risk profiles of two trajectories characterised by persistent wheeze but differentiated by normal or reduced LF differed significantly. Elevated fractional exhaled nitric oxide (FeNO) and allergic sensitisation were highly prevalent in both, but only PEW-RLF was significantly associated with perinatal and early-life factors/exposures (prematurity; lower gestational age: RRRs [95% CI] 2.21 [1.49–3.28], low birth weight: 2.60 [1.47–4.60]: and exposure to smoking during gestation: 2.00 [1.49–2.63]). Two low lung function trajectories (with and without symptoms; PEW-RLF and NIFW-RLF) had similar LF impairment, but divergent clinical and risk factor profiles. PEW-RLF was associated with high rates of asthma diagnosis, high FeNO, bronchodilator reversibility, and family history of atopy. In contrast, those in NIFW-RLF trajectory had no elevation in inflammatory biomarkers and low prevalence of airway hyperreactivity, and were characterised by much higher rates of prenatal tobacco smoke exposure, and greater active adolescent smoking (2.06 [1.41–3.01]), and higher body fat mass in adolescence (1.02 [1.01–1.03], p = 0.01) with no difference in birth weight or preterm birth. Replication analyses in independent cohorts (n = 3388) were consistent with the discovery.

**Interpretation:**

The disconnect between symptoms and lung function, along with the differences in risk profiles, has important implications for respiratory health intervention strategies.

**Funding:**

UK MRC grant MR/S025340/1.

**Research in context**

**Evidence before this study**

Studies of wheeze phenotypes show that although persistent wheezers tend to have lower peak lung function, many retain normal spirometric indices, while some individuals with reduced lung function do not report persistent wheeze. This highlights discordance between symptom and lung function trajectories. However, these outcomes are typically modelled separately, limiting understanding of their joint development. To date, no studies have applied a true joint modelling approach to simultaneously characterise longitudinal wheeze and lung function trajectories, potentially overlooking important discordant trajectories.

**Added value of this study**

This study applies group-based multi-trajectory modelling to jointly characterise wheeze and lung function from childhood to early adulthood across multiple cohorts, identifying six distinct phenotypes. More than half of persistent wheezers maintained normal lung function, whereas approximately 10% of the overall population had persistently reduced lung function despite little or no wheeze. Despite comparable levels of atopy and type 2 inflammation markers, only the persistent wheeze with reduced lung function (PEW-RLF) trajectory was associated with early-life factors, including prematurity, lower birth weight, and prenatal and postnatal smoke exposure. In contrast, the never or infrequent wheeze with reduced lung function (NIFW-RLF) trajectory was characterised by minimal symptoms, low bronchodilator response, low airway hyperreactivity, low atopy, and higher adolescent smoking and body fat mass. This clinically “silent” phenotype may be missed by symptom-based approaches.

**Implications of all the available evidence**

These findings show that joint modelling of wheeze and lung function provides a more comprehensive understanding of respiratory disease heterogeneity than approaches based on single outcomes. Identification of discordant trajectories, particularly individuals with persistently reduced lung function despite minimal symptoms, has important clinical and public health implications. Such individuals may not be detected through symptom-based screening but may be at increased risk of impaired lung growth and future chronic respiratory disease. The results emphasise the role of early-life factors and modifiable exposures, including tobacco smoke and obesity, and support integrating lung function assessment into clinical and epidemiological practice.

## Introduction

Childhood wheezing is common and may be the first clinically recognised manifestation of respiratory morbidity, including asthma.^1^ It is caused by diverse pathophysiological processes,^2^ and consequently not all wheezing follows the same trajectory or carries the same long-term implications.^1^ A substantial effort has been devoted to understanding the heterogeneity of childhood wheezing.^3^ Studies which modelled wheeze longitudinally have shown that lung function in early adulthood is lower in all wheeze phenotypes/clusters than in children who never wheezed, with those with persistent wheezing having the lowest peak lung function.^4^^,^^5^ This is important, as impaired peak lung function is a marker of increased risk for chronic obstructive pulmonary disease,^6^ early all-cause mortality,^7^ and early-onset cardiovascular, respiratory, and metabolic comorbidities.^8^ Parallel to data-driven approaches to disaggregate wheeze, several studies have explored lung function trajectories from childhood to early adulthood and consistently reported an association between childhood asthma/wheezing and low lung function trajectories.^9^, ^10^, ^11^

However, although children with persistent wheezing have on average the lowest peak lung function, the majority of persistent wheezers have normal spirometric indices.^4^^,^^5^ Similarly, among individuals in the persistently low FEV_1_/FVC trajectory, only a third also belong to the persistent wheeze phenotype.^10^ Interestingly, 5–6% of individuals with above-average lung function trajectory had persistent wheezing.^10^ This suggests a degree of discordance between developmental profiles of symptoms and lung function. However, even though wheeze/asthma and lung function are related,^12^ these outcomes are usually modelled separately, limiting our understanding of how they evolve jointly over time. Modelling of single features may overlook subgroups with discordant patterns, such as those who wheeze persistently despite preserved lung function, or those with early airway impairment in the absence of symptoms. Joint longitudinal analyses can identify such discordant phenotypes; for example, analyses in the Urban Environment and Childhood Asthma (URECA) cohort combined longitudinal patterns of wheeze, atopy, and lung function to identify a phenotype characterised by frequent wheeze without atopy and reduced lung function in early childhood, although trajectories in that study were derived for individual markers and then combined to define phenotypes.^13^

We hypothesised that joint modelling of symptoms and lung function may identify previously unrecognised subgroups with distinct developmental pathways, risk profiles, and clinical implications, and uncover subgroups that may require different types of intervention. To address our hypothesis, we used longitudinal data from childhood through early adulthood in four unselected birth cohorts to define and characterise trajectories of wheeze and lung function.

## Methods

### Study populations

We used data from four population-based birth cohorts: The Avon Longitudinal Study of Parents and Children (ALSPAC^14^, ^15^, ^16^), the Manchester Asthma and Allergy Study (MAAS^17^), the Isle of Wight (IOW^18^) from the UK Study Team for Early Life Asthma Research (STELAR) consortium,^19^ and the Swedish Child, Allergy, Milieu, Stockholm, Epidemiological (BAMSE) cohort.^20^ Further details are provided in the Appendix (pp 3 and 4).

### Ethics statement

Ethics approval was obtained for all participating cohorts. The ALSPAC study was approved by the ALSPAC Ethics and Law Committee and Local Research Ethics Committees (IRB00003312). The MAAS cohort received approval from local research ethics committees, including the North West–Greater Manchester East Research Ethics Committee (e.g. 14/NW/1309). The Isle of Wight cohort received approval from the Isle of Wight Local Research Ethics Committee (recruitment, 1, 2, and 4 years) and the National Research Ethics Service Committee South Central–Southampton B (10 and 18 years; 06/Q1701/34). The BAMSE study was approved by the Stockholm Ethical Review Board (2016/1380-31/2). Written informed consent was obtained from parents and/or study participants, as appropriate, in all cohorts.

#### Discovery and replication

We used ALSPAC for the discovery analysis. We conducted two replication analyses. In the first, we used pooled data from two UK cohorts (MAAS and IOW). Details on data pooling are shown in Table S1 (Appendix, p 4). For the second replication, we used BAMSE.

### Data sources

Sex, race, and ethnicity data were obtained via parental report. Parentally- and self-reported symptoms, environmental exposures and demographic and early life factors were ascertained longitudinally using validated questionnaires (Appendix, p 5).

***Lung function:*** We conducted spirometry according to American Thoracic Society/European Respiratory Society (ATS/ERS) guidelines^21^^,^^22^ at ages 8, 15, and 24 in ALSPAC; 8, 16, and 20 in MAAS; 10, 18, and 26 in IOW; 8, 16, and 24 in BAMSE (Appendix; pp 5 and 6). FEV_1_ and FVC were recorded. The assessment time points and sample sizes for wheeze and spirometry in each cohort are shown in Table S2 (Appendix, p 6). This analysis includes participants with at least two spirometry and three wheeze assessments.

***Bronchodilator reversibility (BDR)*** was assessed after administration of 400 μg of salbutamol in ALSPAC, MAAS, and BAMSE, and 600 μg in IOW (ALSPAC at age 15; MAAS at ages 5, 11 and 16; IOW at ages 18 and 26; and BAMSE at age 24 years; Appendix, p 6).

***Airway hyperreactivity (AHR)*** was measured using a rapid methacholine test in ALSPAC between the ages of 8 and 9 years,^23^^,^^24^ in MAAS at ages 8, 11, and 20 years, and in IOW at ages 10 and 18 years (Appendix, pp 6 and 7).

***Airway inflammation:*** FeNO was measured in ALSPAC at age 15, in MAAS at ages 5, 11, and 20, in IOW at age 26, and in BAMSE at ages 16 and 24 years (Appendix, pp 7 and 8). Blood eosinophils were available in ALSPAC at age 24.

***Allergic sensitisation*** was assessed using skin prick tests in ALSPAC at age 8, at 6 time points between ages 3 and 20 years in MAAS, and at 5 time points between ages 1 and 18 years in IOW (Appendix, pp 7 and 8), and by specific IgE at ages 8, 16 and 24 in BAMSE.

### Statistical analysis

Definitions of all variables used in the analysis are provided in the Appendix (pp 9 and 10). To identify trajectory classes based on joint modelling of current wheeze and FEV_1_/FVC (% predicted),^25^ we used group-based multi-trajectory modelling^26^ (details in the Appendix, pp 11 and 12). Both features were analysed simultaneously within a cohesive modelling structure to account for their potential interdependence. The main difference between the commonly used univariate group-based trajectory modelling (GBTM) and multivariate GBTM is that in the latter, each trajectory is a representation of the temporal course of multiple outcomes jointly.^26^ Models with an increasing number of classes were run (from 1 to 20) using the SAS procedure PROC TRAJ.^27^^,^^28^ The final model and the optimal number of trajectories were selected using a Fit-Criteria Assessment Plots (FCAP),^29^ which provide eight goodness-of-fit and model adequacy criteria (Appendix pp 11 and 12).

Figure S1 shows missing data patterns for wheeze and spirometry (Appendix p 13). We did not impute missing data as our modelling approach handles these directly^30^ (Appendix p 12). We conducted two sensitivity analyses to check how sensitive our model is to missing data (Appendix, p 14). Weighted kappa (κ)^31^ and the Rand index^32^ were calculated to measure the agreement between trajectory allocation based on complete and incomplete data.

Variables included in the association analyses are listed in the Appendix (p 14) and Table S3. We considered demographics, early-life factors and environmental exposures. Active smoking was ascertained from early adolescence to early adulthood. Body fat mass was derived from dual-energy X-ray absorptiometry scans.^33^ We investigated the association of these factors and trajectories using weighted multinomial logistic regression. The posterior probability of trajectory membership was included as a weight to reflect the assignment uncertainty. Results are reported as relative risk ratios (RRRs) with 95% CIs.

### Role of the funding source

Sponsors had no role in the study design, data collection, analysis, interpretation, or report writing. The co-authors in charge of the analyses had access to the dataset, and all co-authors shared final responsibility for publication.

## Results

### Characteristics of study populations

**Fig. 1:**
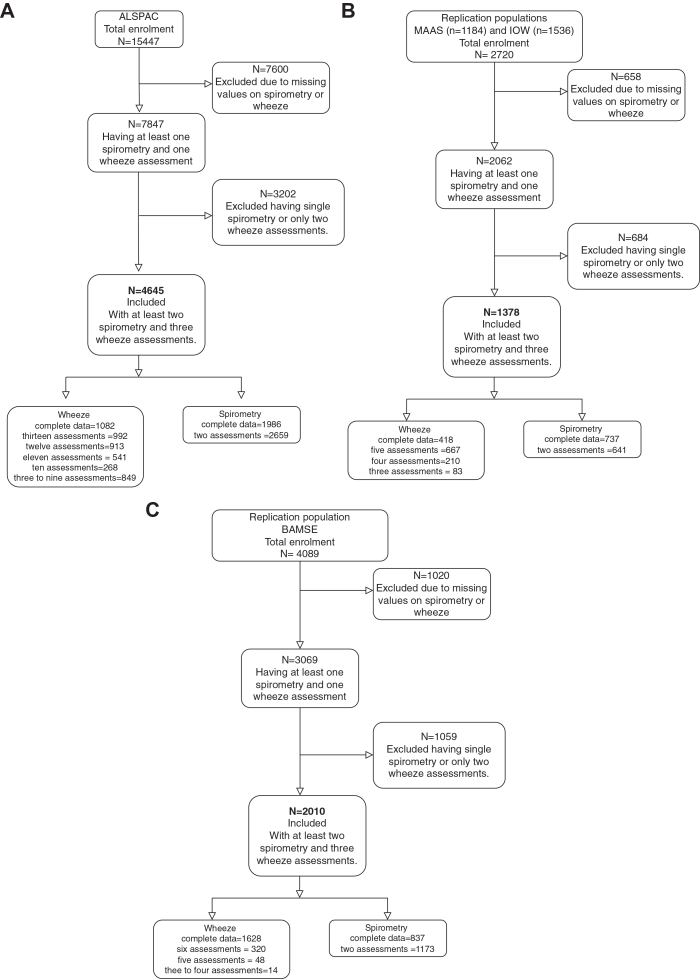
Participant flow: A) Discovery population (ALSPAC); B) Replication 1 (MAAS and IoW); C) Replication 2 (BAMSE).

Participant flow is shown in Fig. 1. In the discovery analysis, we included 4645 subjects. For the first replication, we included 1378 (578 MAAS, 800 IOW), and for the second 2010 participants from BAMSE. Demographic characteristics of study populations are shown in Table S4 (Appendix, p 16). Cohorts were comparable in sex, gestational age, and maternal age. Parental asthma was higher in MAAS; maternal smoking was lower in MAAS and BAMSE. Wheeze in the first year of life was lower in IOW and BAMSE.

### Discovery population: trajectories of current wheeze and FEV_1_/FVC

**Fig. 2:**
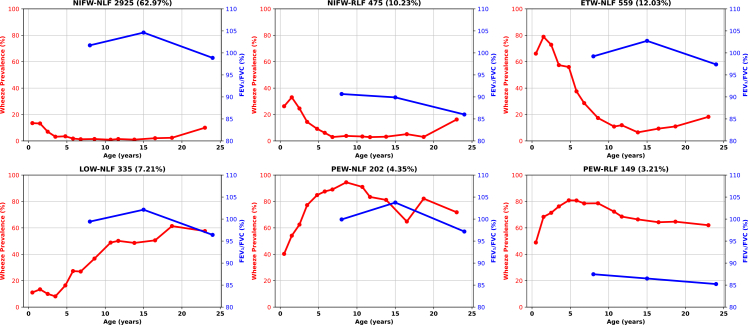
Change in the current wheeze prevalence and mean FEV_1_/FVC GLI percent predicted values with age, by wheeze and FEV_1_/FVC joint trajectories, using data from the discovery population (n = 4645). Each panel presents a trajectory group illustrating the parallel evolution of wheeze prevalence (%) (red left y-axis) and mean FEV_1_/FVC GLI percent predicted (blue, right y-axis). Trajectories were derived by joint modelling of wheeze and lung function (FEV_1_/FVC), i.e., both features were modelled together to capture the developmental patterns of symptoms and lung function. NIFW, never/infrequent wheeze; ETW, early transient wheeze; LOW, late onset wheeze; PEW, persistent wheeze; NLF, normal lung function; RLF, reduced lung function. Reduced lung function trajectories represent relatively lower FEV_1_/FVC values compared with other trajectory groups and do not necessarily indicate values below the lower limit of normal.

In the discovery population (ALSPAC), a six-class model provided an optimal balance among goodness-of-fit, model adequacy criteria, and clinical interpretation. Details about the model selection are provided in the Appendix (p 17), supported by Figures S2 and S3 (Appendix, pp 17 and 18). The average posterior probability of class membership was ∼84% (and the lowest was ∼80%), showing high confidence in the class assignment. Fig. 2 and Figure S4 show changes in the prevalence of current wheeze and mean FEV_1_/FVC with age across the six trajectories. Based on the patterns of current wheeze and FEV_1_/FVC over time, we labelled identified trajectories as: (1) Never/Infrequent wheezing with normal lung function (NIFW-NLF: 2925/4645 [63.0%]); (2) Never/Infrequent wheezing with reduced lung function (NIFW-RLF: 475/4645 [10.2%]); (3) Early Transient wheezing with normal lung function (ETW-NLF: 559/4645 [12.0%]); (4) Late-Onset wheezing with normal lung function (LOW-NLF: 335/4645 [7.2%]); (5) Persistent wheezing with normal lung function (PEW-NLF: 202/4645 [4.4%]); and (6) Persistent Wheezing with reduced lung function (PEW-RLF: 149/4645 [3.2%]). Current wheeze and FEV_1_/FVC at different ages across the six trajectories are presented in Tables S5 and S6, Appendix, pp 20 and 21. The reduced lung function trajectories were defined relative to other trajectory groups and represent consistently lower FEV_1_/FVC values over time rather than values necessarily below the lower limit of normal.

Participants assigned to the NW-NLF trajectory had very low wheeze prevalence from infancy to age 24 (∼1–2% in the school age) and consistently high LF (FEV_1_/FVC 101.7% at 8 years, 98.9% at 24 years). Those in the NIFW-RLF trajectory had slightly higher proportion of early wheeze which resolved by age 7 (12/407 [3.0%] at age 6.7), but persistently low LF (FEV_1_/FVC 90.7% at 8 years, 86.0% at age 24). The ETW-NLF trajectory was characterised by the high prevalence of early wheeze (403/511 [78.9%] at 1.5 years) that declined sharply to 30/462 (∼6.5%) in adolescence, and constantly normal LF. Those in the LOW-NLF had increasing prevalence of wheeze from age 5 years onward, peaking in adolescence (108/176 [61.4%] at age 18.7) with preserved LF (99.4% at 8 years, 96.4% at age 24). Participants assigned to PEW-NLF trajectory had persistently high prevalence of wheeze (>70% at most ages), but stable and normal FEV_1_/FVC. In contrast, those in PEW-RLF trajectory followed a similar wheeze pattern but with consistently low FEV_1_/FVC (87.5% at 8 years to 85.2% at 24 years).

Patterns of other spirometry indices (FEV_1_ and FEF_25–75_) across trajectories were consistent with those observed for FEV_1_/FVC (Table S6, Appendix p 21).

#### Sensitivity analyses

We first ran modelling among 1964 individuals with spirometry at all three and wheeze assessments on at least five time points, followed by analysis in 1737 individuals with spirometry at all three time points and wheeze assessments on at least ten time points. The model selection criteria plots are shown in the appendix (Figure S5, p 22). We observed almost identical six-trajectory optimal solutions (Figure S6, Appendix, p 23). Both analyses revealed strong agreement in trajectory allocation, with weighted kappa values of ∼0.96 and adjusted Rand index values of ∼0.93 (Tables S7 and S8; Appendix p 24).

### Characteristics of the six trajectories

#### Asthma diagnosis, wheeze severity and severe cough

**Fig. 3:**
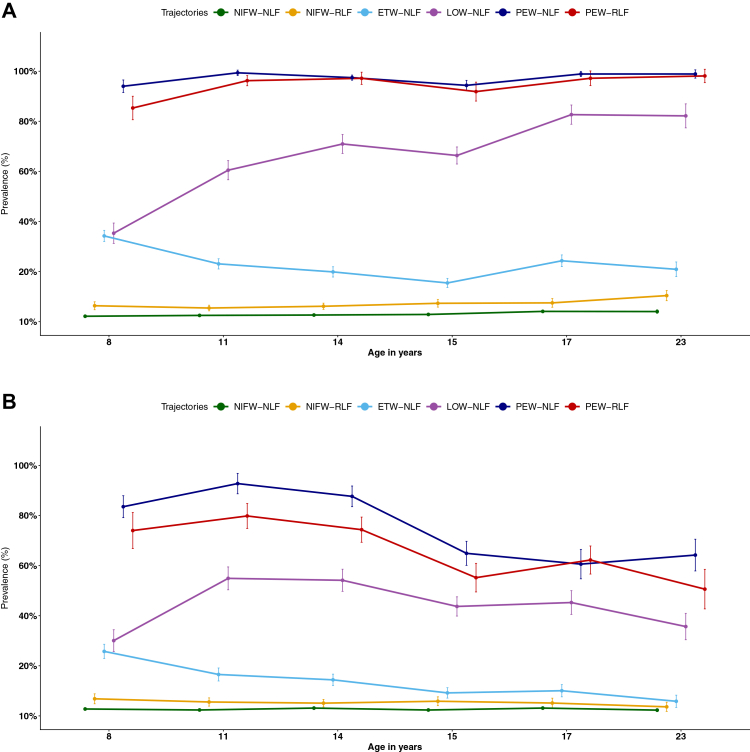
Prevalence of asthma diagnosis and medication use across six joint trajectories of wheeze and FEV_1_/FVC, discovery population. Panel A: Prevalence of asthma diagnosis with 95% CIs from childhood to early adulthood, by joint wheeze and FEV_1_/FVC trajectories. Panel B: Prevalence of asthma medication use with 95% CIs over the same time points, by joint wheeze and FEV_1_/FVC trajectories. Each point represents the estimated prevalence, with vertical lines indicating 95% CIs. To enhance visual clarity, horizontal jitter and separate colours were applied by trajectory. NIFW, never/infrequent wheeze; ETW, early transient wheeze; LOW, late onset wheeze; PEW, persistent wheeze; NLF, normal lung function; RLF, reduced lung function. Current asthma diagnosis: Presence of two of the following three features: 1) Current wheeze; 2) Current use of asthma medication; 3) Physician-diagnosed asthma ever.

Fig. 3 shows the prevalence of asthma diagnosis and medication use across the six trajectories; both were significantly more common in trajectories characterised by persistent wheeze. However, there was no difference in asthma diagnosis and medication use between persistent wheezers with reduced and normal LF.

The distribution of wheeze frequency, severity, and cough requiring a doctor visit across the six trajectories is shown in the appendix (Figure S7, p 25). As expected, a greater proportion of participants with PEW patterns reported frequent wheeze (≥3 episodes in the past 12 months) compared to transient and LOW patterns. However, there was no difference in wheeze frequency between persistent wheezers with reduced and normal LF. Similarly, we observed no differences in severe symptoms (wheezing that disrupted sleep and interfered with speech) between PEW-NLF and PEW-RLF; Figure S7; Appendix, p 25). Furthermore, the prevalence of cough requiring a doctor visit was comparatively higher among PEW-NLF.

#### BDR, AHR, airway inflammation, allergic sensitisation, and eosinophils

The proportions of participants with BDR, AHR, airway inflammation, and allergic sensitisation across the trajectories are shown in Table 1, while their corresponding continuous measurements are presented as box plots in Figure S8 (Appendix, p 26). Among trajectories characterised by the presence of wheezing, the highest proportion of participants with BDR, AHR and elevated FeNO was observed in the PEW-RLF. PEW-RLF also had a high prevalence of allergic sensitisation, but numerically lower than PEW-NLF. Elevated FeNO, BHR and allergic sensitisation were also observed in PEW-NLF and LOW-NLF. Notably, compared to NIFW-RLF, PEW-RLF showed markedly higher FeNO (≥35 ppb; 42/58 [72.41%] vs. 53/169 [31.36%]), greater BDR (36/101 [35.64%] vs. 52/320 [16.25%]), and higher AHR (21/72 [29.2%] vs. 16/284 [5.6%]). ETW-NLF had a modest increase in BDR and AHR, and no significant elevation in FeNO or sensitisation. Blood eosinophils showed a pattern similar to FeNO, with differences across groups primarily driven by wheeze (Appendix, Figure S9, p 26).

**Table 1:** Associations of trajectories with FeNO (n = 1761), BDR (n = 3225) at 14–15 years, airways hyperresponsiveness (AHR) at age 8 (n = 2873), and allergic sensitisation at age 8 (n = 2857) from the discovery population.

	FeNO ≥35 ppb at 14–15 years		BDR >12% at 15 years		AHR at age 8		Sensitisation at age 8	
	n/N (%)	RRR (95% CI)	n/N (%)	RRR (95% CI)	n/N (%)	RRR (95% CI)	n/N (%)	RRR (95% CI)
NIFW-NLF	262/1093 (23.97%)	1 (reference)	99/2038 (4.86%)	1 (reference)	38/1904 (2.00%)	1 (reference)	237/1774 (13.36%)	1 (reference)
NIFW-RLF	53/169 (31.36%)	1.49 (1.04–2.15)	52/320 (16.25%)	4.31 (3.01–6.18)	16/284 (5.63%)	2.93 (1.61–5.33)	43/281 (15.30%)	1.17 (0.82–1.67)
ETW-NLF	50/200 (25.00%)	1.01 (0.69–1.45)	32/388 (8.25%)	1.86 (1.21–2.84)	21/339 (6.19%)	3.24 (1.88–5.99)	71/333 (21.32%)	1.76 (1.31–2.36)
LOW-NLF	81/128 (63.28%)	5.52 (3.71–8.21)	29/242 (11.98%)	2.69 (1.71–4.26)	18/183 (9.84%)	5.36 (2.99–9.59)	101/221 (45.70%)	5.46 (4.05–7.35)
PEW-NLF	52/78 (66.67%)	6.32 (3.83–10.43)	15/136 (11.03%)	2.34 (1.29–4.23)	19/91 (20.88%)	12.96 (7.12–23.59)	97/145 (66.90%)	13.10 (9.03–19.01)
PEW-RLF	42/58 (72.41%)	9.21 (4.99–16.98)	36/101 (35.64%)	11.82 (7.49–18.63)	21/72 (29.17%)	20.25 (11.08–36.89)	58/103 (56.31%)	8.36 (5.53–12.63)

RRRs were estimated from multinomial logistic regression models and represent exponentiated model coefficients.

Sensitisation: any positive SPT > 3 mm (grass, house dust mites, cat, egg, peanuts, and nuts). AHR: airway hyperreactivity is defined as a >20% fall in FEV_1_ at ≤ 6.1 μmol methacholine. BDR: Bronchodilator reversibility is defined as a >12% increase in FEV_1_ after administration of 400 μg of salbutamol.

NIFW, never/infrequent wheeze; ETW, early transient wheeze; LOW, late onset wheeze; PEW, persistent wheeze; NLF, normal lung function; RLF, reduced lung function; RRR, relative risk ratios.

### Associates of different trajectories

**Fig. 4:**
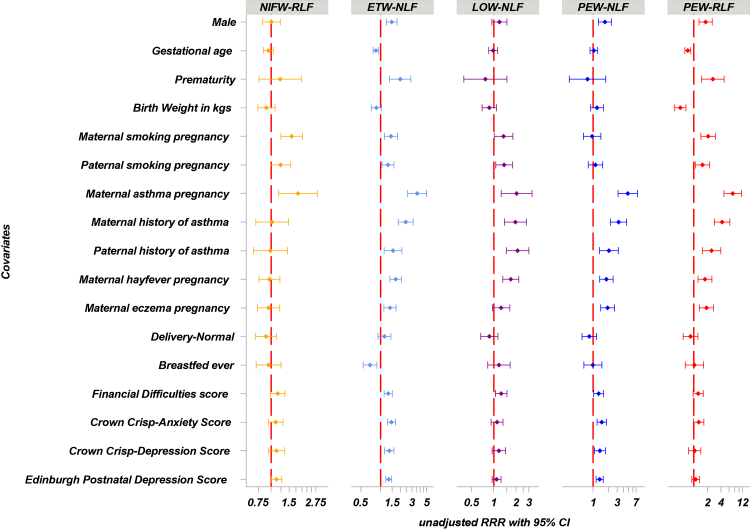
Early life and parental risk factors associated with trajectories; NIFW-NL as a reference. Data are unadjusted relative risk ratios (Wald 95% CI) from the discovery population (ALSPAC). For birth weight, the relative risk ratio is per one kilogramme increase; for gestation age, the relative risk ratio is per two-week increase; for financial difficulty score, anxiety score, and depression score, the relative risk ratios are per a five-unit increase. For categorical variables, the relative risk ratios are for “yes”, with “no” as a reference. RRR: relative risk ratio. Financial difficulties score at 32 weeks of gestation. Anxiety and depression scores at 18 weeks of gestation. Breastfed ever: by 15 months of age. Different x-axis scales were used across panels to enhance visibility, as sample sizes varied between trajectories. RRRs are plotted on a log scale to allow for clearer comparison across a wide range of effect sizes. NIFW, never/infrequent wheeze; ETW, early transient wheeze; LOW, late onset wheeze; PEW, persistent wheeze; NLF, normal lung function; RLF, reduced lung function; gest: gestation.

The results of weighted multinomial logistic regression models (using the NIFW-NLF trajectory as a reference) are shown in Fig. 4; the Adjusted RRRs with 95% CIs are presented in Table S9 (Appendix, p 27). Male sex was associated with a higher relative risk of ETW-NLF trajectory membership, as well as both PEW trajectories. Prematurity increased the relative risk of ETW-NLF and PEW-RLF. An increase in birthweight significantly decreases the relative risk of joining PEW-RLF.

Maternal smoking during pregnancy was associated with a higher relative risk of ETW-NLF, NIFW-RLF, LOW-NLF, and PEW-RLF, but not PEW-NLF. Paternal smoking showed a similar association pattern. Maternal asthma significantly increased the relative risk for all trajectories compared to NIFW-NLF, with the strongest association with PEW-RLF. Maternal hay fever and eczema followed similar association patterns, with the strongest association observed with PEW-RLF and PEW-NLF, with no significant associations with NIFW-RLF.

Psychosocial and socioeconomic factors showed selective associations. Higher maternal anxiety and depression scores (Crown-Crisp index) were significantly associated with ETW-NLF and PEW-NLF. Edinburgh Postnatal Depression scores were only linked to ETW-NLF (1.06 [1.04–1.08], p < 0.001) and PEW-NLF (1.06 [1.03–1.09], p < 0.001). A very similar association pattern was observed after adjustment.

**Fig. 5:**
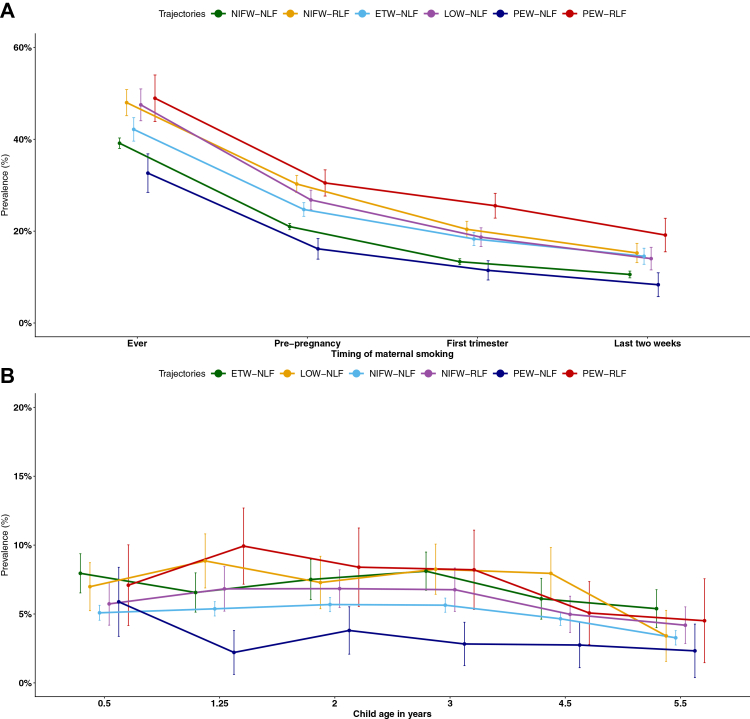
Maternal smoking and higher postnatal tobacco smoke (ETS) exposure across six joint trajectories of wheeze and FEV_1_/FVC, discovery population. Panel A: Prevalence of maternal smoking with 95% CIs at four critical periods: ever smoked, pre-pregnancy, first trimester, and the last two weeks of pregnancy, by joint wheeze and FEV_1_/FVC trajectories. Panel B: Prevalence of ETS exposure from infancy through early childhood (0.5–5.5 years), by joint wheeze and FEV_1_/FVC trajectories. Points represent prevalence, and error bars indicate 95% confidence intervals. To improve readability, jitter was applied to timepoints, and y-axis scales were adjusted independently for each panel. Higher exposure refers to being exposed for more than 3 h a week. NIFW, never/infrequent wheeze; ETW, early transient wheeze; LOW, late onset wheeze; PEW, persistent wheeze; NLF, normal lung function; RLF, reduced lung function.

Tobacco smoke exposure, both prenatal and postnatal, varied markedly across the trajectories (Fig. 5; Appendix Figure S10, p 28). Children in the PEW-RLF trajectory had the highest exposure. Similarly, children in NIFW-RLF had high maternal smoking and sustained moderate environmental tobacco smoke (ETS) exposure across time. In contrast, children with persistent wheeze but normal lung function (PEW-NLF) had the lowest maternal smoking rates of all trajectory groups.

Prevalence of active smoking, along with the relative risk ratios, is shown in Figure S11 (Appendix p29). Active smoking rates were significantly higher in the NIFW-RLF compared to the NIFW-NLF trajectory at all ages from adolescence to early adulthood. Additionally, the NIFW-RLF trajectory consistently showed higher smoking prevalence than most trajectories. For example, at age 24, 59/234 (25.2%) participants in the NIFW-RLF trajectory were smoking, compared to 210/1268 (16.6%) in the NIFW-NLF trajectory.

Among all trajectories, higher body fat mass in adolescence significantly increased the risk of membership only in the NIFW-RLF trajectory, compared to NIFW-NLF (Appendix Figure S12, p 30).

### Replication

Detailed modelling summary is given in the Appendix (Figures S13–S15; pp 31–33). An optimal six-trajectory solution, capturing similar developmental patterns, was evident in both replication populations (Figures S16 and S17, Appendix pp 34 and 35).

Airway inflammation across trajectories is presented in Table S10 (Appendix, p. 36). Median FeNO levels were higher at all ages among persistent wheezers, irrespective of lung function, whereas these differences were less apparent at older ages. BDR and AHR across trajectories are shown in Tables S11 and S12 (Appendix, pp 37 and 38). Participants in the PEW-RLF trajectory showed the highest prevalence of both BDR and AHR at all ages. Notably, NIFW-RLF had markedly lower BDR and AHR than PEW-RLF at all ages.

Allergic sensitisation across trajectories is shown in Figure S18 (Appendix, p 38). The proportion of sensitised individuals was the highest in trajectories characterised by PEW and LOW. Patterns of sensitisation were identical among those with infrequent wheeze, regardless of lung function. Severe exacerbations across the trajectories in MAAS are presented in Figure S19 (Appendix, p. 39), with PEW-RLF exhibiting the highest rate.

Tables S13 and S14 (Appendix, pp 40 and 41) show the risk factor analyses for both replication populations, which were consistent with the discovery population.

## Discussion

In this longitudinal analysis in four birth cohorts that tracked participants from early childhood to early adulthood, we identified six distinct trajectories of the developmental patterns of wheezing and lung function. Using a modelling approach that combines symptoms with spirometry data, we uncovered both expected and novel developmental patterns with distinct risk profiles. Among these, we found previously described group characterised by persistent wheezing with reduced lung function and markers of T2 inflammation,^34^ as well as a less recognised group of persistent wheezers with perfectly normal lung function, and those with no or infrequent wheezing that resolved but with sustained lung function impairment. More than half of persistent wheezers had stable and normal lung function from childhood to early adulthood, whereas at the population level, around 10% had either no wheezing or early infrequent wheeze which resolved by school-age, but with sustained lung function deficit to early adulthood. We observed significant differences in the risk profiles associated with these patterns. This disconnect between symptoms and lung function, along with the differences in risk profiles, has important implications for intervention strategies.

Our analysis reveals distinct risk profiles between the two trajectories, characterised by persistent wheeze but differentiated by normal or reduced LF. Elevated FeNO and allergic sensitisation were highly prevalent in both, indicating T2-high inflammation, further supported by higher median blood eosinophil counts. The URECA cohort identified a persistent wheeze phenotype without atopy and with reduced lung function, whereas both persistent wheeze groups in our study showed evidence of type 2 inflammation. This difference may reflect differences in modelling variables, as our trajectories were defined by wheeze and lung function and did not include inflammatory markers. Both persistent wheeze trajectories were strongly associated with male sex and parental history of allergic diseases; only PEW-RLF was significantly associated with perinatal and early environmental exposures, including prematurity, lower gestational age, low birth weight, and exposure to smoking during gestation. These findings align with studies suggesting that structural or developmental insults may contribute to long-term poor LF among symptomatic children,^35^^,^^36^ and may act synergistically with T2 inflammation to lead to LF impairment. This suggests that addressing airway inflammation alone may not suffice to change the trajectory of lung function. Of note, there was no difference in wheeze frequency and severity or troublesome cough between persistent wheezers with reduced and normal LF. However, in MAAS in which information from medical records was available, severe wheeze exacerbation rates were higher among PEW-RLF, adding to the body of evidence that virus infections and exacerbations contribute to early loss in lung function.^37^^,^^38^

Comparison of the two trajectories characters by reduced lung function, PEW-RLF and NIFW-RLF, showed similar levels of LF impairment, but markedly divergent clinical and risk factor profiles. PEW-RLF was associated with high rates of asthma diagnosis, frequent and sometimes severe wheeze into adulthood, elevated FeNO, BDR, and a strong family history of atopy. In contrast, those assigned to NIFW-RLF had either no or only occasional wheeze confined to early childhood, with no elevation in inflammatory biomarkers and low prevalence of AHR in adolescence. Despite this, children in this group had persistently low lung function from age 8 to 24 years, with a declining pattern and low BDR (suggesting structural changes in airways). These children were rarely diagnosed with asthma and would not be captured by symptom-based screening. This symptomatically “silent” trajectory of diminished lung function is clinically important, as suboptimal lung function growth in early life is a major pathway to adult ill health.^6^^,^^8^^,^^10^ In our analysis, the NIFW-RLF trajectory was characterised by much higher rates of prenatal tobacco smoke exposure, but also greater adolescent smoking and higher body fat mass in adolescence (but with no difference in birth weight or preterm birth). These factors are hallmarks of social deprivation, which is linked to higher smoking rates, but also limited access to healthy foods and lower physical activity which contribute to higher obesity rates in adolescence. Our findings support observations that children whose parents smoke are much more likely to take up smoking themselves (https://www.gov.uk/government/news/children-whose-parents-smoke-are-four-times-as-likely-to-take-up-smoking-themselves), and of a cycle of health inequality which is perpetuated across generations. Of note, although values for both reduced lung function trajectories were generally within the normal range, relatively lower lung function trajectories have been associated with an increased risk of chronic respiratory disease later in life.^6^

NIFW-RLF trajectory likely represents a structural, non-inflammatory pathway to airflow limitation, distinct from that associated with T2 inflammation. In contrast, the PEW-RLF trajectory fits a more classical profile of asthma/T2 inflammation progressing to airway remodelling and persistent airflow obstruction, whose persistent symptoms and inflammatory profile point toward the need for early and sustained anti-inflammatory treatment (with a caveat that there is a component of their lung function impairment that is likely unresponsive to anti-T2 therapies).

Maternal anxiety and depression during pregnancy were associated with trajectories showing early or persistent symptoms but normal LF. This is consistent with findings linking prenatal psychological distress to increased wheeze and asthma risk in children.^39^^,^^40^ While some cohorts^41^^,^^42^ have reported modest lung function reductions, these were primarily observed in children exposed to very high levels of maternal stress and reflected a restrictive pattern. Notably, these deficits occurred with a preserved FEV_1_/FVC ratio,^41^^,^^42^ suggesting reduced lung volumes rather than airway obstruction.

Of note, some risk factors, such as maternal asthma, were associated with multiple trajectories, suggesting shared susceptibility across respiratory phenotypes. In contrast, several early-life environmental factors showed differential associations across trajectories, indicating that different exposures may influence distinct wheeze–lung function patterns. This suggests that while some factors increase overall disease risk, modifiable early-life exposures may influence specific respiratory phenotypes and represent potential targets for prevention. Similar findings were reported in the URECA cohort,^13^ where multiple early-life environmental exposures, including maternal stress, depression, tobacco smoke, and indoor environmental exposures, were differentially associated with specific respiratory phenotypes, supporting the importance of early-life environmental influences on respiratory health trajectories.

Our analysis has several strengths. Previous studies have examined longitudinal respiratory phenotypes in early childhood; however, to our knowledge, this is the first study to apply joint trajectory modelling to symptoms and lung function over two decades of follow-up. Spirometry was repeated at three key developmental ages (8, 15, and 24 years), allowing us to assess lung growth and identify failure to attain high peak function. The sample was well-characterised in terms of perinatal exposures, inflammation, adolescent behaviours, and socioeconomic context. These rich data enabled detailed profiling of each trajectory and the identification of previously unrecognised risk patterns.

However, several limitations must be acknowledged. Wheeze was parent or self-reported, possibly subject to recall bias, particularly in later childhood. Although we captured multiple timepoints of lung function, post-bronchodilator measurements were not always available at all follow-ups. Our definition of a ‘normal’ LF trajectory relied on spirometry, particularly pre-bronchodilator FEV_1_, which is a relatively crude index. As such, values within the ‘normal’ range may still conceal underlying pathology. Our findings are based on a European population cohort; replication in other ethnic and geographical groups is warranted. Furthermore, we could not include inflammatory markers or sensitisation in the trajectory derivation because these were available at a single time point in the main discovery cohort. In addition, sensitisation measures differed across replication cohorts, and some trajectory groups were relatively small, which limited more detailed analyses of multiple sensitisation or inflammatory markers across trajectories.

Our findings suggest that similar impaired spirometric outcomes may arise from markedly distinct mechanistic pathways, including genetic factors. Recognising these distinctions is essential to developing targeted prevention and treatment strategies. Our results have important clinical implications. Firstly, they support expanding respiratory surveillance strategies beyond symptom-focused assessments,^43^ as lung function deficits can occur independently of respiratory symptoms and are associated with distinct clinical and environmental risk profiles. Individuals with no or resolved symptoms yet sustained lung function impairment demonstrated a unique risk profile. Second, these findings highlight the potential for early interventions to alter lung function trajectories before symptoms emerge or reappear, and that spirometry should be used as early in life as possible to identify individuals at risk of poor health.^44^ In addition, in clinical care, this should be coupled with screening for social and environmental factors.

## Contributors

A.C., A.U., R.G. G.R, A.S., and J.W.H. conceived and planned the study and wrote the manuscript. A.C., H.A., C.S.M, S.T., J.W.T., A.S, G.R. and E.M. were responsible for the acquisition of the financial support for the project leading to this publication.

A.U., S.F., and R.G. developed and applied statistical, mathematical, and computational techniques to analyse and synthesise data.

A.U., R.G., S.F., and G.W. were responsible for the annotation and maintenance of the research data (including software code for interpreting the data).

A.U., R.G., S.F. and G.W. were responsible for visualisation/data presentation.

A.C., H.A., C.S.M, S.T., J.W.T., L.L., A.S, G.R. and E.M. conducted investigation process, L.L. performed longitudinal lung function measurements in MAAS.

All authors contributed to the interpretation of the results, have read and approved the final version of the manuscript. All authors provided critical feedback and helped shape the research, analysis, and manuscript. A.U., R.G., S.F. and G.W. directly accessed and verified the underlying data reported in the manuscript.

## Data sharing statement

Deidentified participant data and data dictionary are available to others; each cohort has its own policies and procedure to share the data and can be contacted for data request.

## Declaration of interests

All authors have completed the ICMJE uniform disclosure form. Dr Custovic reports personal fees outside the submitted work from Amgen, La Roche-Posay, Reacta Healthcare, OM Pharma, Allergopharma, Thermo Fisher Scientific, and Stallergenes Greer. Clare Murray reports grants from Innovate UK and GSK, lecture fees from Sanofi and GSK, and has served on an advisory board for AstraZeneca. Graham Roberts reports grants from NIHR, NIH, the European Union, and Chiesi; consulting fees from ALK-Abelló and AstraZeneca paid to his institution, lecture fees from ALK-Abelló paid to his institution; a pending patent on house dust mite immunotherapy to prevent asthma; support from Thermo Fisher for sample processing; participation as Chair of the NIHR-funded Trial Steering Committee (SPIROMAX); and a past presidency of BSACI. Other authors declare no conflict of interest.
