# Supplementary material for: Joint modelling of wheeze and lung function from childhood to early adulthood: four population-based birth cohorts
Source: eClinicalMedicine. 2026 May 29;96:103996. doi: 10.1016/j.eclinm.2026.103996 (PMC13240771; doi:10.1016/j.eclinm.2026.103996)
Supplement: Supplementary Material [file mmc1.docx]

**SUPPLEMENTARY APPENDIX**

**Joint Modelling of Wheeze and Lung Function from Childhood to Early Adulthood: Four Population-based Birth Cohorts**

Anhar Ullah^1,2^, Sara Fontanella^1,2^, Raquel Granell^3^, Lesley Lowe^4^, Hasan Arshad^5,6,7^, Clare S Murray^4^, Steve Turner^8,9^, John W Holloway^5,6^, Gang Wang^10,11^, Angela Simpson^4^, Graham Roberts^5,6,7^, Erik Melen^11,12^**,** Adnan Custovic^1,2^

on behalf of STELAR/UNICORN^10^ investigators

^1^National Heart and Lung Institute, Imperial College London, UK

^2^NIHR Imperial Biomedical Research Centre, London, UK

^3^Department of Population Health Sciences, Bristol Medical School, University of Bristol, UK

^4^Division of Infection, Immunity and Respiratory Medicine, School of Biological Sciences, Faculty of Biology, Medicine and Health, University of Manchester, Manchester Academic Health Science Centre, U.K.

^5^Human Development and Health, Faculty of Medicine, University of Southampton, Southampton, U.K.

^6^NIHR Southampton Biomedical Research Centre, University Hospitals Southampton NHS Foundation Trust, Southampton, U.K.

^7^David Hide Asthma and Allergy Research Centre, Isle of Wight, UK

^8^Royal Aberdeen Children's Hospital NHS Grampian Aberdeen, AB25 2ZG, UK

^9^Child Health, University of Aberdeen, Aberdeen, UK

^10^Department of Integrated Traditional Chinese and Western Medicine, West China Hospital, Sichuan University, Sichuan, China

^11^Department of Clinical Science and Education, Karolinska Institutet, Södersjukhuset, Stockholm, Sweden

^12^Sachs’ Children and Youth Hospital, Södersjukhuset, Stockholm, Sweden

**Corresponding author:**

Prof. Adnan Custovic, MD, PhD, FRCP, FMedSci

National Heart and Lung Institute, Imperial College London, St Mary’s Campus Medical School

Norfolk Place, London W2 1PG.

Email: [a.custovic@imperial.ac.uk](mailto:a.custovic@imperial.ac.uk)

Table of Contents

[**SUPPLEMENTARY METHODS** 3](#_Toc210983385)

[**Study design and population** 3](#_Toc210983386)

[**1.** **The Avon Longitudinal Study of Parents and Children (ALSPAC)** 3](#_Toc210983387)

[**2.** **The Manchester Asthma and Allergy Study (MAAS)** 3](#_Toc210983388)

[**3.** **The Isle of Wight (IOW) cohort** 4](#_Toc210983389)

[**4.** **The Swedish Child (Barn), Allergy, Milieu, Stockholm, Epidemiological survey (BAMSE)** 4](#_Toc210983390)

[**5.** **Data pooling for the first replication analysis** 4](#_Toc210983391)

[**Wheeze and Spirometry** 5](#_Toc210983392)

[**1.** **Wheeze** 5](#_Toc210983393)

[**2.** **Spirometry** 5](#_Toc210983394)

[*ALSPAC* 5](#_Toc210983395)

[*MAAS* 5](#_Toc210983396)

[*IOW* 5](#_Toc210983397)

[*BAMSE* 6](#_Toc210983398)

[**Bronchodilator reversibility (BDR), airways hyperreactivity (AHR), Sensitisation and fraction of exhaled nitric oxide** 7](#_Toc210983399)

[**SUPPLEMENTARY STATISTICAL ANALYSIS** 8](#_Toc210983400)

[**1.** **Group-based multi-trajectory modelling** 8](#_Toc210983401)

[***2.*** ***Fit-*Criteria *Assessment Plot (FCAP)*** 9](#_Toc210983402)

[***3.*** ***Definitions of different FCAP criteria*** 9](#_Toc210983403)

[***4.*** ***Missing data*** 10](#_Toc210983404)

[***5.*** ***Sensitivity Analysis:*** 12](#_Toc210983405)

[***6.*** ***Factors used in association analysis with spirometry and wheeze trajectories*** 12](#_Toc210983406)

[***7.*** ***Definitions of demographic, early life, and parental factors*** 13](#_Toc210983407)

[***8.*** ***Definitions of wheeze severity, asthma diagnosis, and asthma medication use*** 13](#_Toc210983408)

[***9.*** ***Definitions of body fat mass and active smoking*** 14](#_Toc210983409)

[**SUPPLEMENTARY RESULTS** 15](#_Toc210983410)

[***Characteristics of study populations*** 15](#_Toc210983411)

[***1.*** ***Final Model Selection in the discovery population:*** 16](#_Toc210983412)

[***2.*** ***Sensitivity Analyses*** 20](#_Toc210983413)

[**REPLICATION POPULATIONS** 29](#_Toc210983414)

[***1.*** ***Final Model selection first replication populations (pooled data from MAAS and IOW):*** 29](#_Toc210983415)

[***2.*** ***Final Model selection second replication populations (BAMSE):*** 31](#_Toc210983416)

[***3.*** ***Trajectories from first replication populations (pooled data from MAAS and IOW):*** 32](#_Toc210983417)

[***4.*** ***Trajectories from the second replication populations (BAMSE):*** 33](#_Toc210983418)

[***5.*** ***Selected association analysis from both replication populations*** 34](#_Toc210983419)

# **SUPPLEMENTARY METHODS**

## **Study design and population**

### **The Avon Longitudinal Study of Parents and Children (ALSPAC)**

ALSPAC is a birth cohort study established in 1991 in Avon, UK^1-3^. Pregnant women with expected delivery dates from 1st April 1991 to 31st December 1992 were invited to participate in the study. The initial number of pregnancies enrolled is 14,541. Of these initial pregnancies, there was a total of 14,676 foetuses, resulting in 14,062 live births and 13,988 children alive at one year of age.

When the oldest children were approximately seven, an attempt was made to bolster the study with eligible cases who had failed to join initially. As a result, when considering variables collected from the age of seven onwards (and potentially abstracted from obstetric notes), there are data available for more than the 14,541 pregnancies mentioned above. The number of new pregnancies not in the initial sample (known as Phase I enrolment) that are currently represented on the built files and reflecting enrolment status at the age of 24 is 913 (456, 262 and 195 recruited during Phases II, III and IV respectively), resulting in an additional 913 children being enrolled. The enrolment phases are described in more detail in the cohort profile paper and its update. The total sample size for analyses using any data collected after age seven is 15,447 pregnancies, resulting in 15,658 foetuses. Of these, 14,901 were alive at one year of age.

Study data were collected and managed using REDCap Research Electronic Data Capture) electronic data capture tools^4^ hosted at the University of Bristol.

Ethical approval for the study was obtained from the ALSPAC Ethics and Law Committee and the Local Research Ethics Committees. Informed consent for the use of data collected via questionnaires and clinics was obtained from participants following the recommendations of the ALSPAC Ethics and Law Committee at the time. The study website contains details of available data through a fully searchable data dictionary and variable search tool: <http://www.bristol.ac.uk/alspac/researchers/our-data/>

### **The Manchester Asthma and Allergy Study (MAAS)**

MAAS is an unselected birth cohort study established in 1995 in Manchester, UK^5^. It comprises a mixed urban-rural population within 50 square miles of South Manchester and Cheshire, located within the maternity catchment area of Wythenshawe and Stepping Hill Hospitals. All pregnant women were screened for eligibility at antenatal visits (8th-10^th^ week of pregnancy). Of the 1499 couples who met the inclusion criteria (≤10 weeks of pregnancy, maternal age ≥18 years, and questionnaire and skin prick data test available for both parents), 288 declined to take part in the study, and 27 were lost to follow-up between recruitment and the birth of a child. A total of 1184 children were born into the study between February 1996 and April 1998. They were followed prospectively for 20 years to date and attended follow-up clinics for assessments, which included lung function measurements, skin prick testing, biological samples (serum, plasma and urine), and questionnaire data collection. The study was approved by the North West – Greater Manchester East Research Ethics Committee.

We capitalized on a unique feature of the health care system in the UK in that, General practitioners (GPs) maintain primary care records of all health care encounters of their patients, including hospital admission and outpatient appointments. A trained paediatrician extracted and transcribed data from GP-held medical records, including AD diagnoses and prescriptions for topical treatments. Timing, type of visit, symptoms, indication and prescriptions for each encounter were noted. A total of 987 participants provided informed consent for medical data collection. We reviewed 925 study participants due to GPs’ lack of response for data collection or participants moving away. Nine of these were partially accessed due to missing paper or electronic records and were excluded.

Data on lower respiratory tract infections (LRTI), hospital admissions, bronchiolitis, and RSV-positive bronchiolitis were extracted from electronic and paper-based primary care medical records, including emergency department admissions and hospital admissions. Age in days at the time of each event was documented63. This data was available from birth to age 8 years.

Atopic sensitisation was ascertained by skin prick testing (SPT) at ages 8, 11, 16, and 20 years.

### **The Isle of Wight (IOW) cohort**

IOW is an unselected birth cohort study established in 1989 on the Isle of Wight, UK^6-8^. After the exclusion of adoptions, perinatal deaths, and refusal for follow-up, written informed consent was obtained from parents to enrol 1,456 newborns (of 1536 born between 1^st^ January 1989 and 28^th^ February 1990). Follow-up assessments were conducted up to 26 years of age to prospectively study the development of asthma and allergic diseases. At each follow-up, validated questionnaires were completed by the parents. Additionally, the Skin Prick Test (SPT) was performed on 980, 1036 and 853 participants at 4, 10 and 18 years of age to check allergic reactions to common allergens. Ethics approvals were obtained from the Isle of Wight Local Research Ethics Committee (now named the National Research Ethics Service, NRES Committee South Central – Southampton B) at recruitment and for the subsequent follow-ups. The IOW research team are grateful to all the participants and their families for their support over the years and also to the many fellow researchers who have contributed to the cohort’s follow-up.

### **The Swedish Child (Barn), Allergy, Milieu, Stockholm, Epidemiological survey (BAMSE)**

The BAMSE cohort is a population-based birth cohort that recruited 4089 Swedish children born between February 1994, and November 1996^9^, the cohort was designed to study risk factors for asthma, allergic diseases, and lung function in childhood, and to study factors of importance for prognosis in individuals with established disease. Questionnaires were completed at recruitment at around 3 months of age. Follow-up questionnaires were answered by parents at ages 1, 2, 4, 8, and 16 years, and by participants at age 24 years. A spirometry test was done at age 24 years. For the present study, we included 1817 participants who had available lung function data at age 24 years.

### **Data pooling for the first replication analysis**

We pooled data from the MAAS and IOW cohorts for replication analysis based on shared time intervals for wheeze and spirometry.

**Table S1:** Data harmonisation for the first replication populations (MAAS and IOW).

| **MAAS** | **IOW** | **Shared time interval** |
| --- | --- | --- |
| **Age in Years** | **Age in Years** | **Age in Years** |
| **Current wheeze** | | |
| 1 | 1 | 1 |
| 3 | 2 | 2-3 |
| 5 | 4 | 4-5 |
| 8 | 10 | 8-10 |
| 16 | 18 | 16-18 |
| 20 | 26 | 20-26 |
| **Spirometry** | | |
| 8 | 10 | 8-10 |
| 16 | 18 | 16-18 |
| 20 | 26 | 20-26 |
| IOW=Isle of Wight. MAAS=Manchester Asthma and Allergy Study. | | |

## **Data sources**

### **Symptoms**

Postal questionnaires were used in ALSPAC, while interviewer-administered questionnaires were employed in MAAS and IOW, available on multiple occasions from infancy to early adulthood. In BAMSE questionnaires on respiratory symptoms and medication were answered at 1, 2, 4, 8, 16, and 24 years of age.

Wheezing reports were available at 14 time points in ALSPAC over 23 years, 6 in MAAS over 20 years, 6 in IOW over 26 years, and 7 in BAMSE over 24 years. Table S2 shows specific time points and sample sizes for wheeze data.

### **Spirometry**

### *ALSPAC*

Spirometry tests were conducted at 8^1/2^, 15 and 24 years according to American Thoracic Society/European Respiratory Society guidelines^10,11^ using a Vitalograph pneumotachograph system with animated incentive software (Spirotrac, Vitaograph, UK) in a dedicated research clinic by trained technicians. Calibration checks were performed with a standard 3L calibration syringe according to the manufacturer’s instructions at the start of each half-day clinic session. Subjects were seated with a nose clip in place and were asked to inhale to total lung capacity (TLC), then instructed to perform a forced expiration, through a mouthpiece, to residual volume (RV). The test was repeated at intervals of 30 seconds until 3 technically acceptable traces were obtained from a maximum of eight attempts. Forced expiratory volume in one second (FEV_1_) and Forced vital capacity (FVC) were recorded and the data expressed as FEV_1_ % predicted and FEV_1_/FVC ratio.

### *MAAS*

Spirometry was performed at ages 8, 11, 16 and 20 years according to American Thoracic Society/European Respiratory Society guidelines^10,11^ using a Lilly pneumotachograph system with animated incentive software (Jaeger, Germany). For home visits, we used a flow turbine spirometer (Micro Medical, UK). Subjects were asked to inhale to total lung capacity (TLC), then instructed to perform a forced expiration, through a mouthpiece, to residual volume (RV). The test was repeated at intervals of 30 seconds until 3 technically acceptable traces were obtained. Forced expiratory volume in one second (FEV_1_) and Forced vital capacity (FVC) were recorded and the data expressed as FEV_1_ % predicted and FEV_1_/FVC ratio. Short-acting β2-agonists were withheld for at least four, and long-acting for at least 24 hours prior to testing. Participants were symptom-free at the time of assessment.

### *IOW*

Pre-bronchodilator lung function tests were conducted at 10, 18, and 26 years of age. Forced vital capacity (FVC), forced expiratory volume in 1 second (FEV1) were measured using a Koko Spirometer and software with a portable desktop device (both PDS Instrumentation, Louisville, KY, USA). Spirometry was performed and evaluated according to the American Thoracic Society (ATS) criteria. The children or adults, respectively, were required to be free of respiratory infection for 2 weeks and not to be taking any oral steroids and were advised to abstain from any β-agonist medication for 6 h and from caffeine intake for at least 4 h.

### *BAMSE*

Lung function testing was performed at 8 years using the 2200 Pulmonary Function Laboratory (Sensormedics, Anaheim, CA, USA) and at 16 and 24 years with the Jaeger MasterScreen-IOS system (Carefusion Technologies, San Diego, CA, USA). For each lung function test, the highest values of forced expiratory volume in 1 second (FEV1) and forced vital capacity (FVC) were recorded

The time points for spirometry and the corresponding sample sizes are given in Table S1.

**Table S2:** Wheeze and spirometry assessments' time points and sample sizes in discovery and replication populations.

| **Current wheeze ALSPAC** | | **Current wheeze MAAS** | | **Current wheeze IOW** | | **Current wheeze BAMSE** | |
| --- | --- | --- | --- | --- | --- | --- | --- |
| **Age in Years** | **Sample size** | **Age in Years** | **Sample size** | **Age in Years** | **Sample size** | **Age in Years** | **Sample size** |
| 0.5 | 4273 | 1 | 565 | 1 | 752 | 1 | 1973 |
| 1.5 | 4245 | 3 | 567 | 2 | 698 | 2 | 1967 |
| 2.5 | 4063 | 5 | 577 | 4 | 708 | 4 | 1975 |
| 3.5 | 4139 | 8 | 581 | 10 | 800 | 8 | 1946 |
| 4.75 | 4070 | 16 | 553 | 18 | 795 | 12 | 1941 |
| 5.75 | 3970 | 20^*^ | 493 | 26^*^ | 699 | 16 | 1942 |
| 6.75 | 3963 |  |  |  |  | 24^*^ | 1862 |
| 8.5 | 3972 |  |  |  |  |  |  |
| 10.67 | 4032 |  |  |  |  |  |  |
| 11.67 | 4051 |  |  |  |  |  |  |
| 13.83 | 3960 |  |  |  |  |  |  |
| 16.5 | 3557 |  |  |  |  |  |  |
| 18.67^*^ | 2282 |  |  |  |  |  |  |
| 23^*^ | 2637 |  |  |  |  |  |  |
| **Lung function: FEV_1_/FVC** | | | | | |  |  |
| **ALSPAC** | | **MAAS** | | **IOW** | | **BAMSE** | |
| **Age in Years** | **Sample size** | **Age in Years** | **Sample size** | **Age in Years** | **Sample size** | **Age in Years** | **Sample size** |
| 8 | 4354 | 8 | 512 | 10 | 752 | 8 | 1491 |
| 15 | 3874 | 16 | 533 | 18 | 748 | 16 | 1743 |
| 24 | 3048 | 20 | 469 | 26 | 511 | 24 | 1621 |
| ALSPAC=Avon Longitudinal Study of Parents and Children. IOW=Isle of Wight. MAAS=Manchester Asthma and Allergy Study. BAMSE=Child (*Barn*), Allergy, Milieu, Stockholm, Epidemiological.  *Self-reported wheeze | | | | | | | |

## **Bronchodilator reversibility (BDR)**

Post-BD lung function was tested 15 minutes after administration of 400 μg of salbutamol in ALSPAC, MAAS, and BAMSE, and 600 μg in IOW. From pre- and post-salbutamol lung function measurements,

**Airways hyperreactivity (AHR)**

In ALSPAC, each participant inhaled three breaths of normal saline from a hand-operated bulb nebuliser (DeVilbiss Co., Hanover, PA). The FEV₁ measured 1 minute later was taken as the baseline for the challenge. At 1-minute intervals, participants then inhaled eight doubling doses of methacholine (0.03–6.1 mmol). FEV₁ was measured 1 minute after each dose, immediately followed by the next dose. The challenge was stopped when FEV₁ decreased by >20% from the postsaline value (defined as AHR= yes) or when the maximum dose (6.1 mmol) was reached (defined as AHR= no)^12^.

In MAAS, AHR was assessed through methacholine challenge with a 5-step protocol performed according to American Thoracic Society guidelines. Quadrupling doses of methacholine (0.0625-16.0 mg/mL) were delivered to subjects through a DeVilbiss 646 nebulizer (Sunrise Medical HHG, Somerset, Pa) and a KoKo dosimeter (Pulmonary Data Services, Doylestown, Pa) calibrated to deliver 0.009 mL per 0.6-second actuation. The predicted FEV_1_ was calculated, and if the measured value was less than 1.0 L or less than 60% of the predicted value, the test was not performed. FEV1 was measured 30 and 90 seconds after 5 inhalations of each dose of methacholine. The challenge was stopped when either a 20% decrease in FEV_1_ was observed or the maximum methacholine concentration had been administered^13^.

In IOW, airways hyperresponsiveness (AHR) was assessed using a methacholine challenge test conducted with a Koko dosimeter (PDS Instrumentation, USA), operated with a compressed air source at 8 L/min and a nebuliser output of 0.8 L/min. The procedure began with the inhalation of 0.9% saline to establish a baseline. One minute after saline inhalation, spirometry was performed to record baseline lung function, specifically FEV₁. Following the baseline measurement, participants inhaled increasing concentrations of methacholine, beginning at 0.0625 mg/mL and doubling up to a maximum of 16 mg/mL. After each dose, FEV₁ was measured using standard spirometry. The test continued until a ≥20% decline in FEV₁ from the post-saline baseline was observed or the maximum dose was reached^14^.

**Airway** **inflammation**

In ALSPAC, FeNO values were measured online at a constant flow of 50 mL/s according to European Respiratory Society and American Thoracic Society guidelines by using a Sievers NOA-280i nitric oxide analyzer (GE Analytical Instruments, Boulder, Colo). Feno measurements were done before spirometric measurements. Children were requested to omit their inhaled corticosteroids, if applicable, 48 hours before their visit to the clinic.

In MAAS, FeNO was measured at ages 11, 16 and 18-20 years using either a chemiluminescence analyser (NIOX, Aerocrine, Sweden) or electrochemical analyser (NIOX Mino, Aerocrine, Sweden NIOX), changed on the 4th May 2012. The devices gave comparable results in previous studies.

In IOW, participants had their FeNO measured (Niox mino, Aerocrine AB, Solna, Sweden) according to ATS guidelines. A biofeedback mechanism was used to maintain the expiratory flow rate at 50 ml/s and subjects exhaled against resistance to prevent upper airway contamination. Measurements were made in a standardised manner with the subject standing without a nose clip; FeNO was measured prior to spirometric testing^14^. Participants were rescheduled if they had clinical symptoms consistent with either current infection or a recent (within two weeks) asthma exacerbation or had required antibiotics or oral steroids in the preceding two weeks.

In BAMSE, FeNO was measured with a chemiluminescence analyzer (EcoMedics Exhalyzer® CLD 88sp with Denox 88, Eco Medics, Duernten, Switzerland). The procedure was performed in accordance with published guidelines.[11](https://pmc.ncbi.nlm.nih.gov/articles/PMC12314734/#pai70154-bib-0011)Mean exhalation flow rate was 50 mL/s ± 10% during the NO plateau. The mean value of two measurements within 10% of the mean was used for analysis. FeNO was defined as the mean of these values expressed in ppb. The analyser was calibrated using a standard NO calibration gas (Air Liquide Deutschland GmbH, Krefeld, Germany).

**Sensitisation***:* The atopic status of the children was determined (at an annual clinic when the children were 7–8 years of age in ALSPAC^15^, at ages 3, 5, 8, 11, 16 and 20 in MAAS, and at age 1,2,4,10 and 18 in IOW) by skin prick test responses to a panel of up to 12 common allergens including house dust mite, mixed grasses and cat. Sensitisation to one of these three allergens has been shown to identify 95% of all sensitised children in this population. A positive response was defined as a mean weal diameter of >3 mm with an absent response to the negative control solution, and atopy was defined as a positive response to one or more of house dust mite, cat or grass pollen. Due to the small sample size for IOW, we only included data at ages 4,10 and 18 years.

In BAMSE, at ages 8,16, and 24 years, airborne allergen sensitisation was assessed to a mix of common airborne allergens with Phadiatop® (ImmunoCAP System; ThermoFisher, Uppsala, Sweden) and a positive test was defined as allergen-specific IgE ≥0·35 kUA/L.

**Exacerbations in MAAS**

*Severe exacerbation of wheeze/asthma:* Defined from medical records as either receipt of oral corticosteroids (OCS) for at least 3 days, or emergency department visit because of asthma/wheeze requiring systemic corticosteroids, or hospital admission.^16,17^ We ascertained the age in days of each exacerbation to provide an accurate account of each episode.

**Definitions of variables**

**Current wheeze:** Current wheeze was defined as a positive response to either the question “Has your child had wheezing in the last 12 months?” or “Has your child had wheezing with whistling in the last 12 months” in ALSPAC and MAAS and IOW cohorts to the question “Has your child had wheezing or whistling in the chest in the last 12 months?”. In BAMSE, current wheeze was defined as one or more episodes of wheezing in the last 12 months.

**Wheeze frequency:** Number of times the child has/had wheezing and whistling in the past year.

**Wheeze severity:** Defined based on response to questions “Wheezing ever been severe enough to limit the respondent's speech to only one or two words at a time between breaths in the past 12 months” and “Sleep was disturbed due to wheezing in the past year”.

**Current asthma diagnosis:** Presence of two of the following three features: 1) Current wheeze; 2) Current use of asthma medication; 3) Physician-diagnosed asthma ever.

**Asthma medications:** Based on the question “Child prescribed asthma medication in the past year/past 12 months”.

**Cough requiring a doctor's visit:** Based on the question “Child had cough” (“Did not have”, “Yes, & saw a doctor”, “Yes, but did not see doctor”).

**Percent predicted FEV_1_/FVC ratio:** The per cent predicted FEV_1_/FVC ratio for each individual and at each time point was derived using Global Lung Initiative (GLI) reference equations^18^.

**Bronchodilator reversibility (BDR):** We calculated the percentage change of FEV_1_ from baseline as follows:

([Post-FEV_1_]−[Pre-FEV_1_])/(Pre-FEV_1_×100%)

BDR was defined a 12% or greater increase in FEV_1_

## **Airways hyperreactivity (AHR):** AHR was defined as >20% fall in FEV_1_ at ≤ 6.1 μmol methacholine in ALSPAC, and ≤ 16 mg/ml methacholine in MAAS and IOW.

## **Airway inflammation:** FeNO>35 ppb was used to define the likely eosinophilic airway inflammation^19^

## **Allergic sensitisation:** Defined as a wheal diameter of 3mm greater than the negative control to one or more allergens in ALSPAC, MAAS, and IOW. In BAMSE, it is defined as allergen-specific IgE ≥0·35 kUA/L/L.

### **Demographic, early life, and parental factors**

**Prematurity:** Defined as gestational age < 37 weeks.

**Maternal and paternal smoking:** Defined based on the response given to the question “Do you (or does your partner) smoke?” administered during pregnancy or 1st year of the study child.

**Parental history of asthma, eczema and hay fever:** Assessed through questionnaires and was defined based on the responses to the question “Have you ever had asthma/eczema/hay fever?”.

**Financial difficulties score^20^:** The financial difficulties score was constructed of a series of five questions. The mother was asked to rate on a scale from zero to three how difficult it is currently to afford food, clothes, heating, rent/mortgage and other things considered essential for the child, with higher scores indicating more difficulty.

**Crown Crisp-Anxiety Score^21^:** Maternal prenatal anxiety was measured using the Crown Crisp–Anxiety Score, derived from the eight-item anxiety subscale of the Crown Crisp Experiential Index (CCEI), a validated self-report questionnaire. Items are rated on a 4-point scale (‘very often’ to ‘never’), with higher scores reflecting greater anxiety. In the ALSPAC cohort, this scale has previously demonstrated good reliability, with internal consistency coefficients above 0.80 during pregnancy^22^.

**Crown Crisp-Depression Score^21^:** Maternal prenatal depression was measured using the Crown Crisp–Depression Score, derived from the depression subscale of the Crown Crisp Experiential Index (CCEI), a validated self-report questionnaire. Items are rated on a 4-point scale (‘very often’ to ‘never’), with higher scores reflecting greater depressive symptoms.

**The Edinburgh Postnatal Depression Scale (EPDS)^21^:** Prenatal depressive symptoms were measured using the Edinburgh Postnatal Depression Scale (EPDS), a validated 10-item self-report questionnaire. Items ask women to rate how they felt over the past week (e.g., ‘Things have been getting on top of me’), with higher scores indicating greater depressive symptoms. The EPDS has demonstrated good reliability in pregnancy, with an internal consistency coefficient of 0.87^22^.

**Environmental tobacco smoking exposure (ETS):** Based on the questions “Time child spends in smokers' room” or “Passive smoke exposure”. We created a binary variable, higher ETS exposure, if the child was exposed to ETS for three or more hours a week.

## **SUPPLEMENTARY STATISTICAL ANALYSIS**

### **Group-based multi-trajectory modelling**

Group-based multi-trajectory modelling is a generalisation of group-based trajectory modelling (GBTM)^23^. GBTM identify clusters of individuals following similar trajectories of a single marker of interest, such as wheeze. The main interest is the pattern of outcome (wheeze) conditional on age (or time of visit). Let $Y_{i}$ denotes the individual $i$ longitudinal sequence of wheeze measurements, and the vector ${age}_{i}$represent the age of the $i$ -th individual when each of those measurements was recorded. The group-based trajectory model assumes that the population distribution of trajectories arises from a finite mixture of unknown order $J$. The likelihood of each individual $i$, conditional on the number of groups $J$, may be written as

$$P\left( Y_{i}|{Age}_{i} \right)=\sum_{j=1}^{J} \pi_{j}.P(Y_{i}|{Age}_{i},j;\beta_{j})$$

Where $\pi_{j}$ is the probability of membership in the group $j$, and the conditional distribution of $Y_{i}$ given membership in $j$ is indexed by the unknown parameter vector $\beta_{j}$, which, among other things, determines the shape of the group-specific trajectory.

In the multi-trajectory model framework, each of the $j$ trajectory groups are defined by a set of trajectories for multiple outcomes. Let $K$ denote the number of such outcomes with the random vector $Y_{i}^{k}$representing individual $i$ longitudinal sequence of measurements of the $k-th$ outcome and let $P_{k}(Y_{i}^{k}|{Age}_{i},j,\beta_{j}^{k})$ denote the distribution of that vector conditional on the group $j$ and the unknown parameter vector $\beta_{j}^{k}$. The likelihood of each individual $i$, conditional on the number of groups $j$, may be written as

$$P\left( Y_{i}^{1},Y_{i}^{2},\ldots,Y_{i}^{K} \right)=\sum_{j=1}^{J} \pi_{j}\left[ \prod_{k=1}^{K} P_{k}(Y_{i}^{k}|{Age}_{i,}j,\beta_{j}^{k}) \right]$$

### ***Fit-*Criteria *Assessment Plot (FCAP)***

The Fit-Criteria Assessment Plot (FCAP)^24^ is an automated visual display of several fit criteria recorded when fitting multi-GBTM for different and increasing numbers of classes. Readers are referred to the original publication for a closer description of this plot (Klijn et al. 2017). In a gist, The FCAP combines eight goodness-of-fit and model-adequacy criteria in compact graphs for several user-stipulated varying k’s (i.e., number of classes). Details about all the fit criteria are given below.

### ***Definitions of different FCAP criteria***

**BIC:** SAS define the BIC as

$$BIC=\log\left( L \right)-0.5plog(N)$$

Where$L$is the model’s (maximised) likelihood, $N$ is the sample size and $p$is the number of parameters in the model. Both the AIC (the same expression given for BIC, but without $N$) and BIC penalises for the number of parameters used in the model, while BIC additionally penalises more severely by considering the sample size. A lower value of BIC indicates better model fitting.

**Average posterior probability of assignment (APPA):** The APPA for a group is the mean of the posterior probability of assignment **(PPA)** of individuals assigned to this group. A threshold of 0.70 is considered an acceptable limit.

**Odds of correct classification (OCC):** The OCC compares the odds of correctly classifying subjects into a group $j$ based on the maximum probability classification rule (APPA for class$j$), correcting for the OCC based on random assignment. For each group $j$, OCC is defined as

$$OCC_{j}=\frac{\frac{{APPA}_{j}}{(1-{APPA}_{j})}}{\frac{\hat{\pi}}{(1-\hat{\pi})}}$$

Higher OCCs indicate a better fitting model, and an OCC above 5.0 for all groups shows good assignment accuracy.

**Mismatch:** The mismatch is the difference between the estimated group probability $\hat{\pi}_{j}$ and the actual proportion of the sample assigned to a group $j$ ${(P}_{j}=\frac{N_{j}}{N}$).

**SD-GMP:** The standard deviation of group membership probability (SD-GMP) calculates the standard deviation of assignment probability per group based on the individuals assigned to that group. This measure can be expected to have lower values when models have more groups. Lower SD-GMP scores indicate a better-fitting model.

**Size of smallest group:** A final criterion is the percentage of individuals estimated to be assigned to the smallest group, thus the smallest $\pi_{j}$. A cut-off of 1% is often applied for this. This threshold, however, may be adjusted to the sample size, with smaller samples requiring a larger minimum percentage to warrant enough members in the smallest latent class.

FCAP reports four subplots: the models AIC, BIC, and L are displayed in the first subplot. APPA, mismatch, and SD are shown in the second subplot, OCC is shown in the third subplot, and $\pi_{j}$ in the fourth and last subplot. Two lines are displayed for some criteria (APPA, OCC, mismatch and SD). The first one is the mean value of all groups for that criterion. The second value is denoted by either a plus or a minus sign. This indicates that the given value is that of the group with, respectively, the highest or lowest score for that criterion, i.e. worst case scenario. The complementary information of the worst-case scenario is a reassurance that model fit and adequacy indices are kept within acceptable boundaries for all selected classes.

### ***Missing data***

Missing data were observed in spirometry and wheeze at different ages. The missing data pattern for wheeze and spirometry is given in Figure S1. Overall, ~19.0% of spirometry data and ~18.0% of wheeze were missing in the discovery population. The corresponding values in the first replication populations were 15.6% and 6.8%, and the second replication population were 19.5% and 3.3%. The highest missingness was observed at later ages, i.e., early adulthood. We did not impute missing data on spirometry or wheeze because the model used to derive trajectories was capable of handling missing data. PROC TRAJ uses the maximum likelihood method to estimate parameters, including group sizes and shapes of trajectories. Subjects with missing data are included in the analysis, but only available data for each subject are used^25^.

**Figure S1:** Missing data pattern (A) in the discovery population (ALSPAC, n=4645), (B) first replication populations (MAAS and IOW, n=1378), and (C) second replication population. (BAMSE, n=2010)

wheeze (left); spirometry (right)


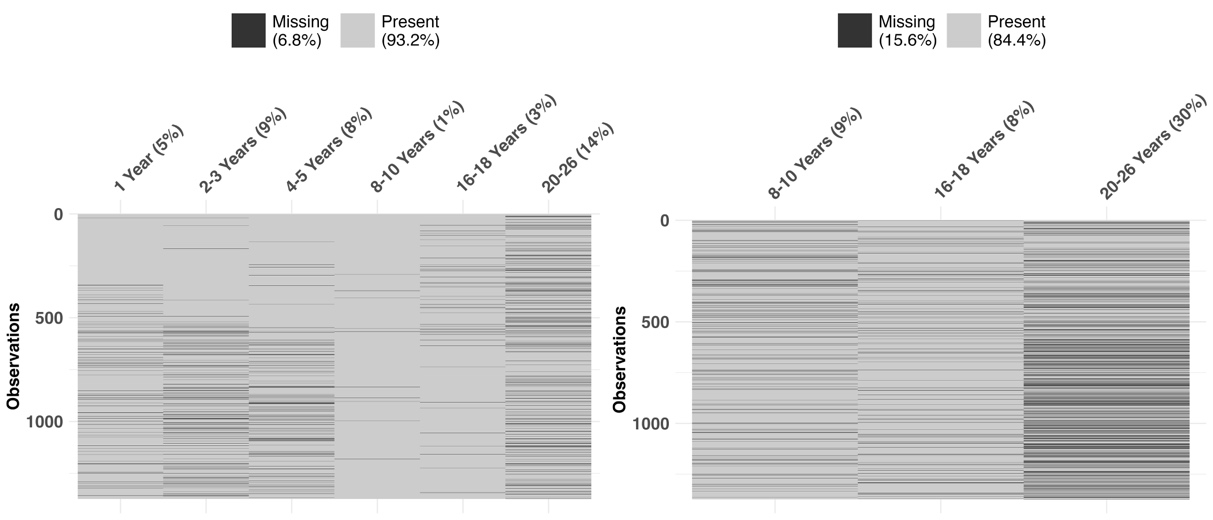

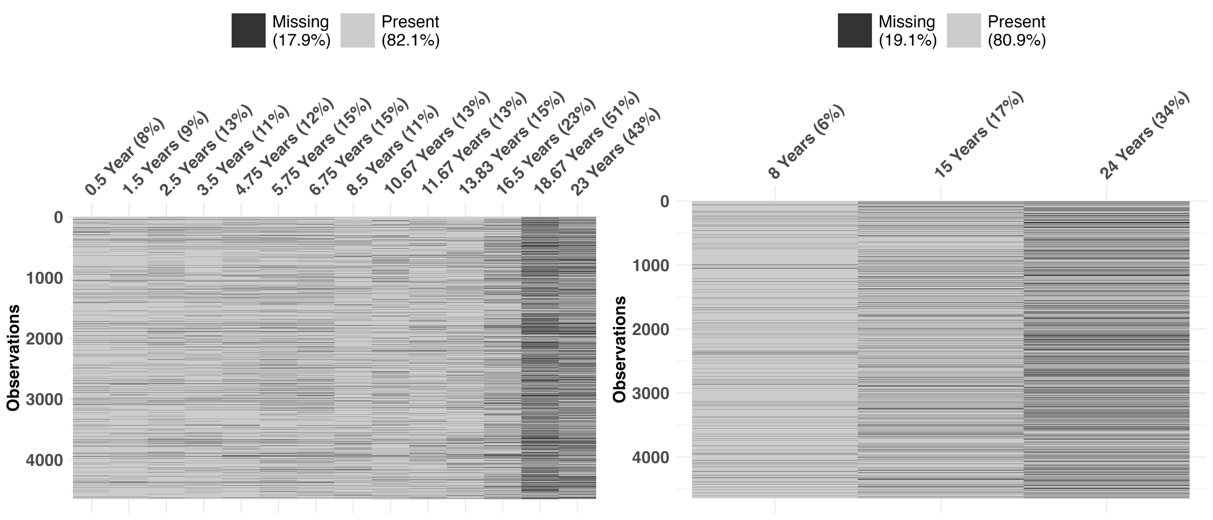

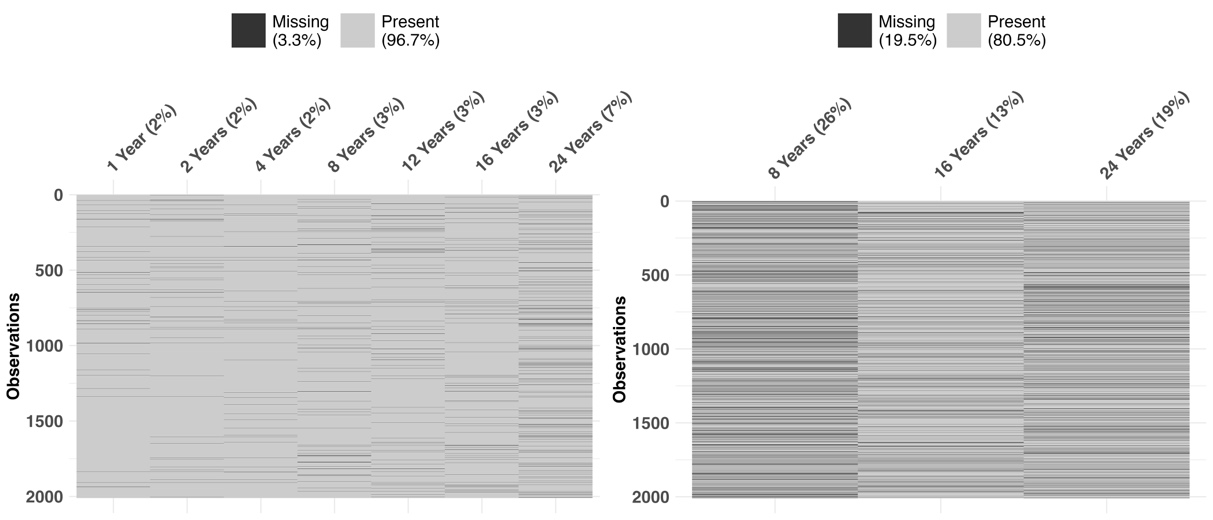


### ***Sensitivity Analyses:***

In the primary analysis for the discovery population, we included participants with at least two spirometry and three wheeze assessments. We conducted two sensitivity analyses to assess the sensitivity of our trajectory derivation model to missing data. In the first sensitivity analysis, we included participants with complete spirometry data and at least 5 wheeze assessments. In the second sensitivity analysis, we included participants with complete spirometry data and at least 10 wheeze assessments. To quantify the agreement between trajectory allocation based on incomplete and complete data, we used the weighted kappa (K^26^ and the Rand index^27^. Kappa and Rand Index are measures of agreement or similarity. The Rand Index is more specifically used to measure the similarity between two data clustering techniques by considering all sample pairs and counting pairs assigned to the same or different clusters.

### ***Variables used in association analysis with current wheeze and spirometry trajectories***

We included the following variables in the association analysis in the discovery population:

#### **Demographic, early life, and parental factors:**

1. Sex
2. Gestational age (weeks)
3. Prematurity
4. Birth weight (in grams)
5. Maternal smoking during pregnancy
6. Partner smoking-pregnancy
7. Maternal asthma (during pregnancy and history)
8. Paternal history of asthma
9. Maternal Hay fever during pregnancy
10. Maternal eczema during pregnancy
11. Ever breastfed by 15 months
12. Delivery type
13. Financial Difficulties Score at 32 weeks of gestation
14. Crown Crisp-Anxiety Score at 18 weeks of gestation
15. Crown Crisp-Depression Score at 18 weeks of gestation
16. Edinburgh Postnatal Depression at 18 weeks of gestation
17. Environmental tobacco smoking exposure

#### **Wheeze severity, asthma diagnosis, asthma medication and cough:**

1. Wheeze frequency at multiple times from six months to 23 years.
2. Wheeze disturbs sleep at multiple times from 6 to 23 years.
3. Wheeze disturbs speech at multiple times from 6 to 23 years.
4. Asthma diagnosis at multiple times from 8 to 23 years.
5. Asthma medication at multiple times from 8 to 23 years.
6. A cough needs a visit to a doctor available at multiple times from 3 to 14 years.

#### **Body fat mass and self-smoking:**

1. Body fat mass at ages 10,12,14, and 15 years.
2. Daily smoking at ages 16,18,20, and 24 years

To investigate how time-variant factors such as symptom severity, clinical outcomes, and environmental tobacco smoke (ETS) exposure vary across trajectories, we present the age-specific prevalence with 95% CIs for each trajectory group.

| **Variable** | **Level** | **N = 4645** | **%** |
| --- | --- | --- | --- |
| Sex | Male | 2136.0 | 46.0 |
|  | Female | 2509.0 | 54.0 |
| Prematurity | Yes | 212.00 | 4.8 |
|  | No | 4250.0 | 95.2 |
| Mother smoking during pregnancy | Yes | 877.00 | 19.7 |
|  | No | 3566.0 | 80.3 |
| Partner smoking-pregnancy | Yes | 1259.0 | 29.6 |
|  | No | 3000.0 | 70.4 |
| Mother recent asthma-pregnancy | Yes | 280.00 | 6.5 |
|  | No | 4043.0 | 93.5 |
| Mother asthma-history | Yes | 512.00 | 11.7 |
|  | No | 3853.0 | 88.3 |
| Paternal history of asthma | Yes | 429.00 | 12.9 |
|  | No | 2905.0 | 87.1 |
| Maternal Hay fever during pregnancy | Yes | 1325.0 | 30.4 |
|  | No | 3040.0 | 69.6 |
| Mother eczema-pregnancy | Yes | 1084.0 | 24.8 |
|  | No | 3281.0 | 75.2 |
| Deliver type | Normal/ vaginal | 3263.0 | 74.5 |
|  | Others | 1116.0 | 25.5 |
| Ever breastfed by 15 months | Yes | 3517.0 | 82.9 |
|  | No | 723.00 | 17.1 |
| Gestational age (weeks) | Mean (SD) | 39.49 (1.80) | - |
| Birth weight (in grams) | Mean (SD) | 3.43 (0.53) | - |
| Financial Difficulties Score at 32 weeks of gestation | Mean (SD) | 2.32 (3.17) | - |
| Crown Crisp-Anxiety Score at 18 weeks of gestation | Mean (SD) | 4.56 (3.33) | - |
| Crown Crisp-Depression Score at 18 weeks of gestation | Mean (SD) | 4.11 (2.91) | - |
| Edinburgh Postnatal Depression at 18 weeks of gestation | Mean (SD) | 6.26 (4.49) | - |

**Table S3:** Distribution of risk factor variables included in the main analyses in the discovery cohort. Continuous variables are presented as mean (SD), and categorical variables as n (%).

# **SUPPLEMENTARY RESULTS**

## ***Characteristics of study populations***

**Table S4:** Characteristics of the study populations.

|  | ALSPAC (n=4645) | MAAS (n=578) | IOW (n=800) | BAMSE (n=2010) |
| --- | --- | --- | --- | --- |
| *Sex* |  |  |  |  |
| *Male* | 2136/4645 (46.0%) | 278/578 (48.10%) | 372/800 (46.5%) | 914/2010 (45·5%) |
| *Female* | 2509/4654 (54.0%) | 300/578 (51.90%) | 428/800 (53.5%) | 1096/2010 (54·5%) |
| White European ancestry | 3526/3599 (97.9%) | 556/578 (96·2%) | 800/800 (100%) | 2010/201 (100%) |
| Gestational age, weeks | 39.49 (1.80) | 39·93 (1·57) | 39.93 (1.51) | 39.79 (1.86) |
| Maternal age at pregnancy, years | 29.05 (4.46) | 30·69 (4·80) | 27.11 (5.30) | 30.99 (4.43) |
| Birthweight, centiles | 61.17 (28.69) | 61.91 (28.31) | 56.98 (29.25) | 66.42 (27.04) |
| Parental asthma | 892/4371 (20.41%) | 176/578 (30.45%) | 151/794 (19.02%) | 412/1997 (20.60%) |
| Maternal smoking during pregnancy | 877/4443 (19.74%) | 74/584 (12·7%) | 158/794 (19.9%) | 228/2009 (11.3%) |
| Breastfeeding during first 6 months | 3447/4225 (81·6%) | 419/565 (74·2%) | 604/754 (80·1%) | 1661/1958 (84·8%) |
| Wheeze in the first year of life | 990/4273 (23.17%) | 147/565 (26.0%) | 83/752 (11.0%) | 288/1973 (14.6%) |
| Data are presented as n/N (%) or mean (SD). ALSPAC=Avon Longitudinal Study of Parents and Children. IOW=Isle of Wight. MAAS=Manchester Asthma and Allergy Study. BAMSE=Child (*Barn*), Allergy, Milieu, Stockholm, Epidemiological. | | | | |

## ***Final Model Selection in the discovery population:***

The functional form of the trajectories was determined prior to selecting the number of trajectory groups by comparing linear and quadratic time specifications and selecting the most appropriate shape based on model fit and trajectory plausibility, resulting in linear terms for lung function and quadratic terms for wheeze. We then began by fitting a two-trajectory model with only two underlying groups assumed. We then fit additional models with an increasing number of trajectories up to 20. Model selection was based on a summary diagnostic plot (Figure S2), which presents key evaluation metrics across all models, including minimum average posterior probability (AvePP−), maximum mismatch across classes (Mismatch +), maximum standard deviation of posterior probabilities (SD+), and change in BIC (ΔBIC). This plot was constructed by choosing selected criteria from the comprehensive FCAP plot, which is shown in full in Figure S3. The BIC continuously improved with the increasing number of classes, showing an asymptotic behaviour expected for large sample sizes^24^. Such steady improvement is a well-known problem with this criterion. So, we must look at other criteria for selecting the final model. Based on the minimum average posterior probability (AvePP−), we restrict our candidate models up to 9 classes, as AvePP− remained above the acceptable 0.70 threshold up to the 9-class model but dropped below this level thereafter. Among the candidate models ranging from 2 to 9 latent classes, the 6-class solution was selected as the optimal model. The 6-class model substantially improved BIC (ΔBIC = +176 vs. 5-class), beyond which gains were minimal (ΔBIC = +58 from 6 to 7 classes). Classification diagnostics further supported this choice: the average posterior probability (AvePP) was 0.839, well above the threshold of 0.70, with a mismatch rate of approximately 3% and a moderate standard deviation of posterior probabilities, indicating confident and well-separated group assignments. Although the improvement in BIC after 6 classes was noticeable, more importantly, classification diagnostics, including minimum average posterior probability (AvePP−), Mismatch+, and SD+, began to deteriorate beyond 6 classes. Specifically, mismatch+ and SD+ increased, while AvePP− declined, suggesting that additional classes were less stable and less well-separated. Models with more than six classes introduced complexity without proportional benefit and often split coherent trajectory patterns.

**Figure S2:** Comparison of model fit and classification metrics across trajectory solutions ranging from 2 to 20 classes. The main plot displays the BIC, while secondary axes represent average posterior probability (AvePP), mismatch rate, and the standard deviation (SD) of posterior probabilities. ΔBIC values between successive models are annotated. The 6-class model (highlighted in yellow) was selected based on its optimal trade-off between statistical fit and classification accuracy, with minimal additional benefit observed in more complex models.

**AvePP:** Average posterior probability; **AvePP -:** minimum average posterior probability across classes, **Mismatch +:** maximum mismatch across classes; **SD+:** maximum standard deviation of posterior probability across classes. ΔBIC: difference between successive BIC values.

**
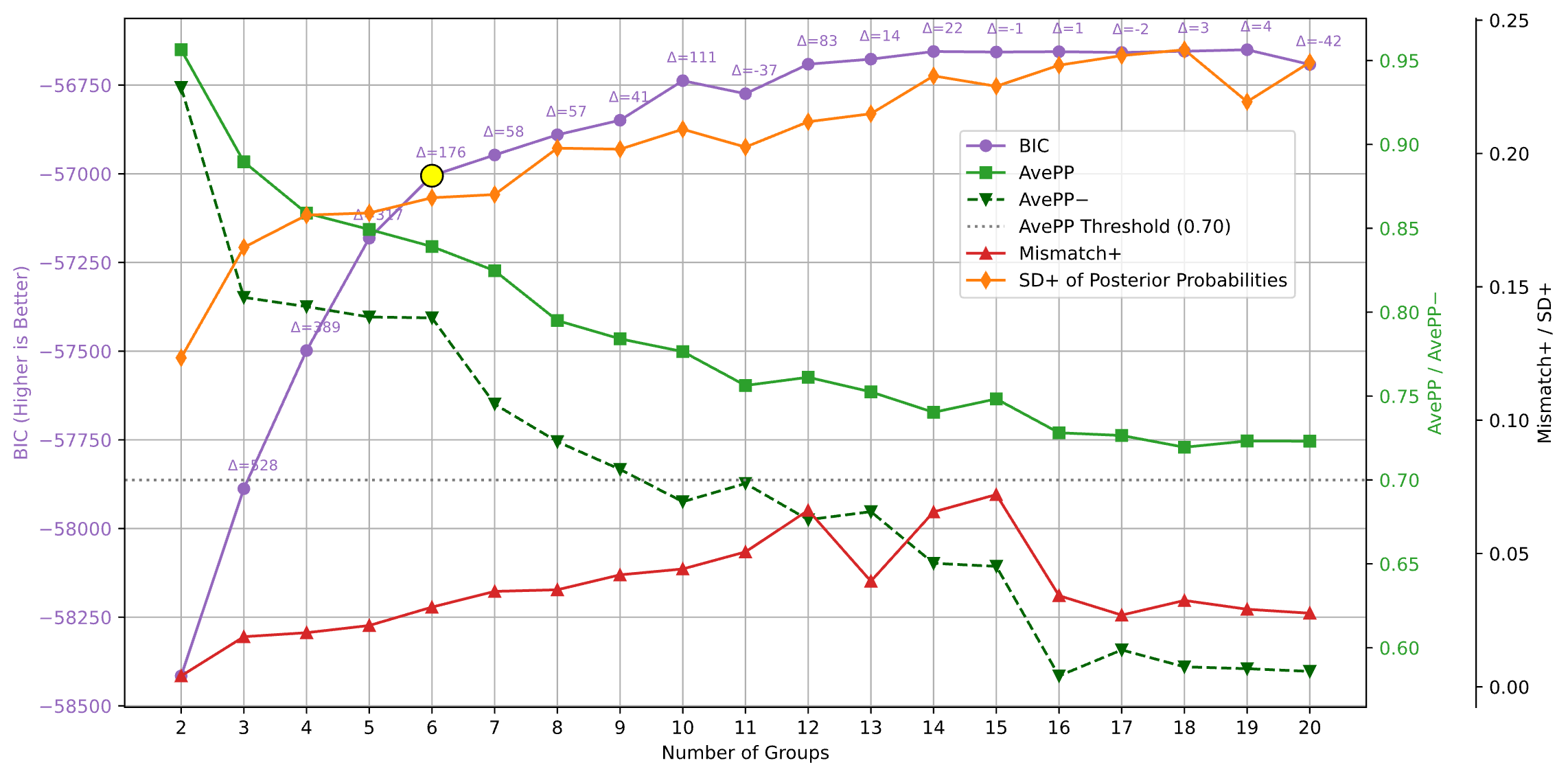
**

**Figure S3:** Fit-Criteria Assessment Plots for discovery population.

1. Akaike's information criterion, Bayesian information criterion and Likelihood.
2. Posterior probability, mismatch and SD
3. Odds of correct classification (5 or above is considered better)
4. Percentage of individuals belonging to the smallest group (1% or above )

A)

B)

C)

D)

**Figure S4:** Change in the current **wheeze prevalence and** mean FEV₁/FVC GLI percent predicted values **with age, by wheeze and** FEV₁/FVC joint trajectories**, using data from the discovery population (n=4645).**

Panel A shows mean FEV₁/FVC (% predicted) across age for each trajectory group, and Panel B shows current wheeze prevalence across age. Points represent means and prevalence, and error bars represent 95% confidence intervals for FEV₁/FVC and current wheeze. Trajectory groups were derived using joint modelling of wheeze and lung function, with both outcomes modelled simultaneously.

NIFW: never/infrequent wheeze; ETW: early transient wheeze; LOW: late onset wheeze; PEW: persistent wheeze; NLF: normal lung function; RLF: reduced lung function

Reduced lung function trajectories represent relatively lower FEV₁/FVC values compared with other trajectory groups and do not necessarily indicate values below the lower limit of normal.

**Table S5:** Number and percentage (n%) of current wheeze at different ages by trajectories. Discovery population.

NIFW: never/infrequent wheeze; ETW: early transient wheeze; LOW: late onset wheeze; PEW: persistent wheeze; NLF: normal lung function; RLF: reduced lung function

| **Age in Years** | **NIFW-NLF** | **NIFW-RLF** | **ETW-NL** | **LOW-NL** | **PEW-NL** | **PEW-RLF** |
| --- | --- | --- | --- | --- | --- | --- |
| 0.5 | 363/2691 (13.49) | 114/434 (26.27) | 334/505 (66.14) | 35/316 (11.08) | 75/186 (40.32) | 69/141 (48.94) |
| 1.5 | 354/2676 (13.23) | 140/424 (33.02) | 403/511 (78.86) | 42/312 (13.46) | 99/183 (54.1) | 95/139 (68.35) |
| 2.5 | 179/261 (6.99) | 101/411 (24.57) | 351/482 (72.82) | 30/300 (10) | 110/176 (62.5) | 95/133 (71.43) |
| 3.5 | 83/2599 (3.19) | 61/423 (14.42) | 282/491 (57.43) | 25/307 (8.14) | 139/180 (77.22) | 106/139 (76.26) |
| 4.75 | 90/2564 (3.51) | 37/403 (9.18) | 272/486 (55.97) | 49/298 (16.44) | 151/178 (84.83) | 114/141 (80.85) |
| 5.75 | 45/2505 (1.8) | 25/407 (6.14) | 173/461 (37.53) | 80/293 (27.3) | 148/169 (87.57) | 109/135 (80.74) |
| 6.75 | 32/2495 (1.28) | 12/405 (2.96) | 132/461 (28.63) | 79/293 (26.96) | 155/174 (89.08) | 106/135 (78.52) |
| 8.5 | 38/2600 (1.46) | 16/422 (3.79) | 85/489 (17.38) | 111/302 (36.75) | 172/182 (94.51) | 103/131 (78.63) |
| 10.67 | 23/2544 (0.9) | 14/416 (3.37) | 51/468 (10.9) | 150/307 (48.86) | 152/167 (91.02) | 94/130 (72.31) |
| 11.67 | 37/2566 (1.44) | 12/415 (2.89) | 57/478 (11.92) | 150/299 (50.17) | 141/169 (83.43) | 85/124 (68.55) |
| 13.83 | 25/2502 (1) | 13/407 (3.19) | 30/462 (6.49) | 140/288 (48.61) | 138/170 (81.18) | 87/131 (66.41) |
| 16.5 | 47/2267 (2.07) | 18/350 (5.14) | 39/418 (9.33) | 134/265 (50.57) | 94/145 (64.83) | 72/112 (64.29) |
| 18.67 | 35/1469 (2.38) | 7/230 (3.04) | 28/255 (10.98) | 108/176 (61.36) | 69/84 (82.14) | 44/68 (64.71) |
| 23 | 170/1699 (10.01) | 45/277 (16.25) | 50/274 (18.25) | 114/198 (57.58) | 79/110 (71.82) | 49/79 (62.03) |

**Table S6:** Mean and standard deviation of Pre-bronchodilator lung function (FEV_1_/FVC, FEV_1_ and FEF- 25- 75) at different ages by trajectories. Discovery population.

NIFW: never/infrequent wheeze; ETW: early transient wheeze; LOW: late onset wheeze; PEW: persistent wheeze; NLF: normal lung function; RLF: reduced lung function

| **Trajectories** | **Sample size** | **Age** | **FEV_1_/FVC**  **Mean (SD)** | **FEV_1_**  **Mean (SD)** | **FEF 25-75**  **Mean (SD)** |
| --- | --- | --- | --- | --- | --- |
| NIFW-NLF | 2925 | Age 8 years | 101.72 (5.42) | 101.22 (11.45) | 103.98 (20.52) |
|  |  | Age 15 years | 104.62 (5.99) | 92.95 (14.90) | 99.27 (21.83) |
|  |  | Age 24 years | 98.87 (5.78) | 96.44 (11.35) | 92.58 (19.85) |
| NIFW-RLF | 475 | Age 8 years | 90.66 (6.57) | 94.22 (11.72) | 72.49 (17.03) |
|  |  | Age 15 years | 89.89 (8.38) | 85.00 (15.39) | 69.55 (16.83) |
|  |  | Age 24 years | 86.01 (6.53) | 87.39 (11.83) | 61.98 (15.21) |
| ETW-NLF | 559 | Age 8 years | 99.22 (6.45) | 98.43 (11.46) | 94.72 (21.48) |
|  |  | Age 15 years | 102.74 (6.64) | 92.02 (14.69) | 94.47 (22.27) |
|  |  | Age 24 years | 97.39 (5.88) | 94.67 (10.66) | 87.89 (18.47) |
| LOW-NLF | 335 | Age 8 years | 99.43 (6.90) | 97.62 (12.15) | 94.07 (20.65) |
|  |  | Age 15 years | 102.13 (6.81) | 90.16 (13.50) | 90.69 (19.04) |
|  |  | Age 24 years | 96.41 (6.37) | 94.67 (11.19) | 87.20 (21.50) |
| PEW-NLF | 202 | Age 8 years | 99.92 (6.76) | 99.01 (12.60) | 95.84 (23.45) |
|  |  | Age 15 years | 103.75 (7.35) | 91.60 (14.91) | 93.57 (21.87) |
|  |  | Age 24 years | 97.17 (6.52) | 93.59 (10.13) | 86.54 (18.96) |
| PEW-RLF | 149 | Age 8 years | 87.47 (7.20) | 89.48 (12.48) | 63.62 (17.13) |
|  |  | Age 15 years | 86.50 (9.86) | 80.86 (16.81) | 61.25 (18.91) |
|  |  | Age 24 years | 85.24 (7.53) | 85.77 (12.04) | 60.21 (14.76) |
| FEF: Forced Expiratory Flow 25-75 (L/Sec) | | | | | |

## ***Sensitivity Analyses***

**Figure S5:** Comparison of model fit and classification metrics across trajectory solutions ranging from 2 to 15 classes. The main plot displays BIC, while secondary axes represent average posterior probability (AvePP), mismatch rate, and the standard deviation (SD) of posterior probabilities. ΔBIC values between successive models are annotated. The 6-class model (highlighted in yellow) was selected based on its optimal trade-off between statistical fit and classification accuracy, with minimal additional benefit observed in more complex models.

**AvePP:** Average posterior probability; **AvePP -:** minimum average posterior probability across classes, **Mismatch +:** maximum mismatch across classes; **SD+:** maximum standard deviation of posterior probability across classes. ΔBIC: difference between successive BIC values.

1. First sensitivity analysis


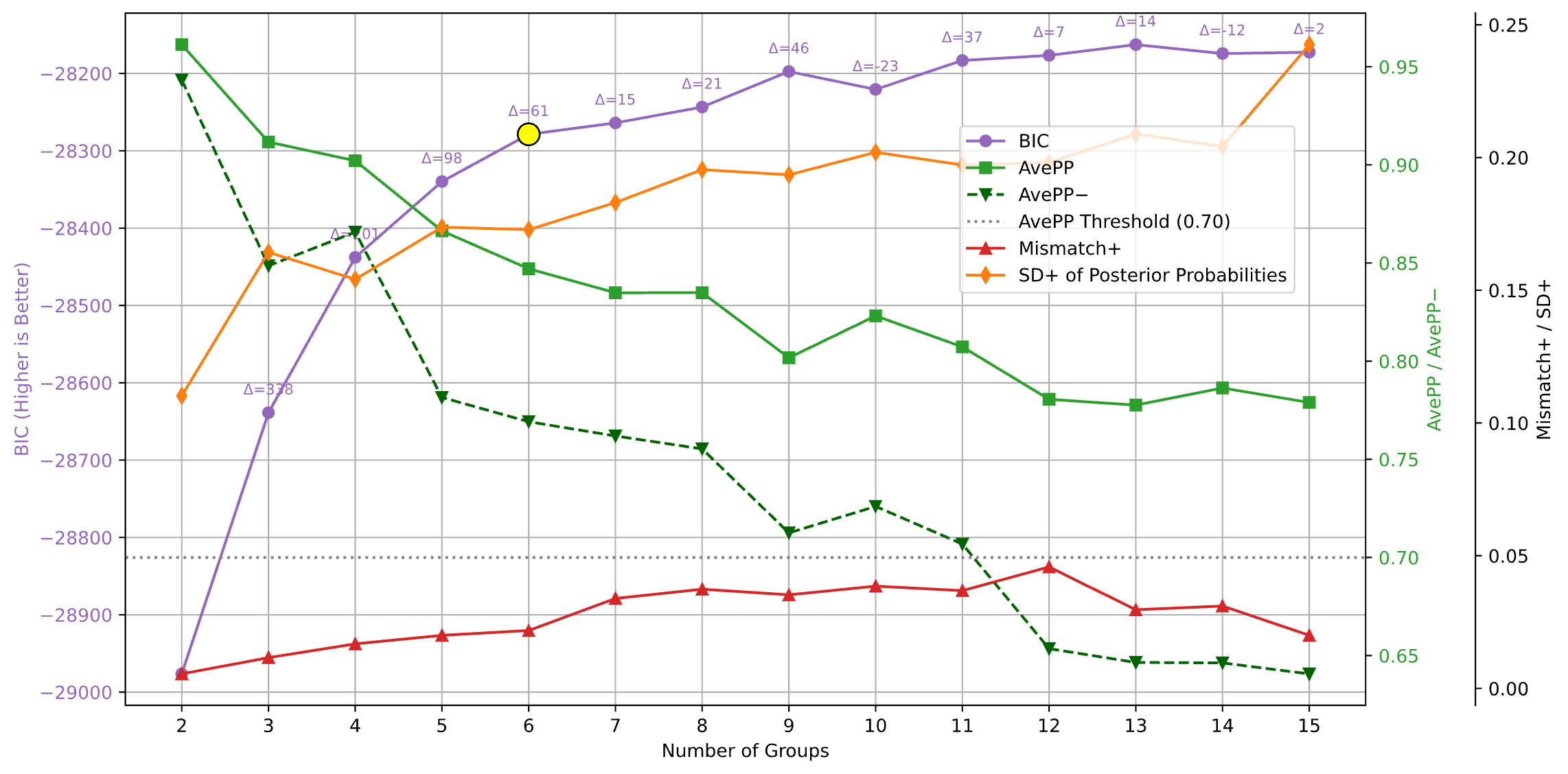


1. Second sensitivity analysis

**
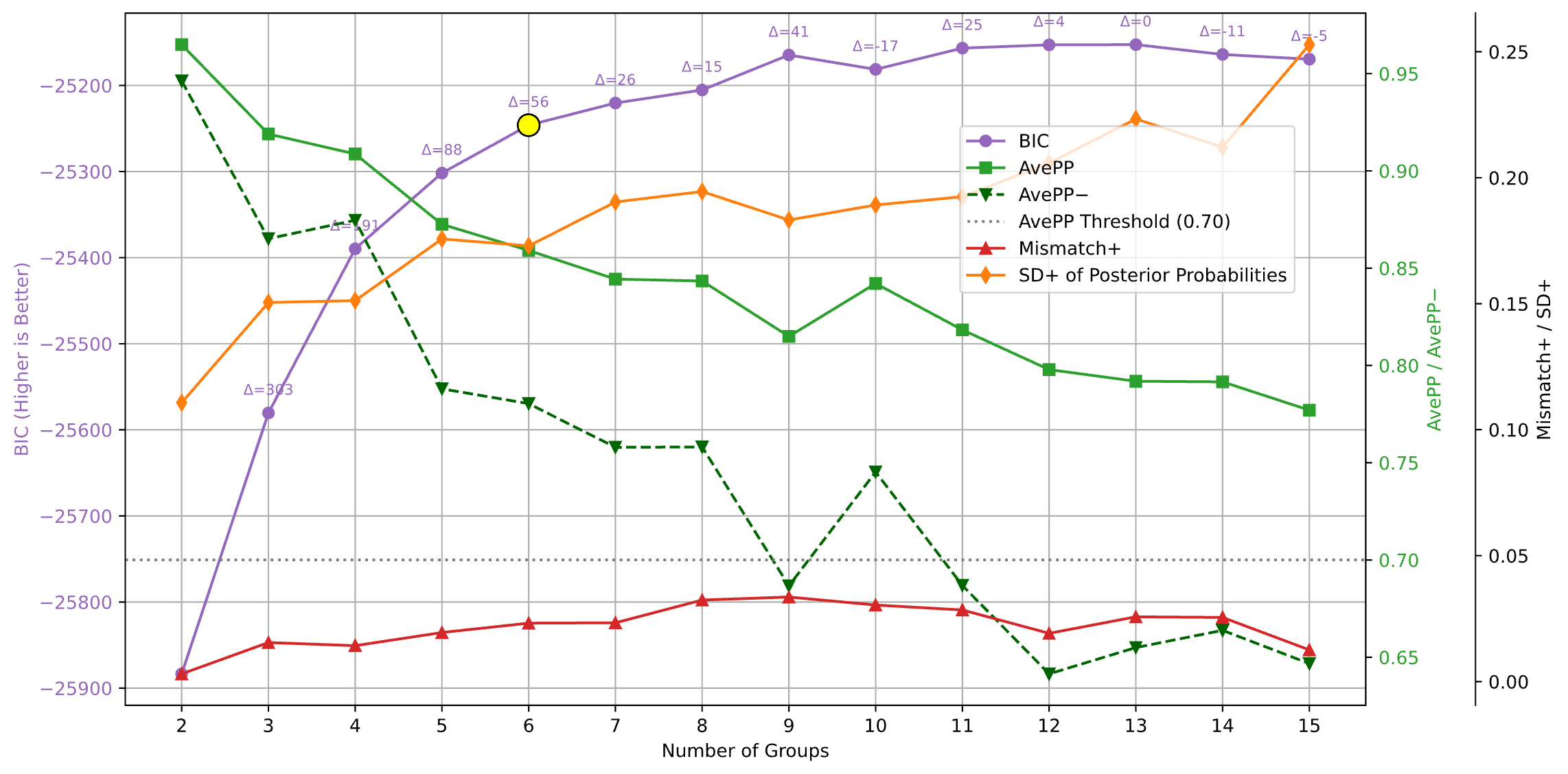
**

**Figure S6:** Change in current wheeze and mean FEV_1_/FVC GLI-percent predicted values with age, by joint trajectories of wheeze and FEV_1_/FVC: (top) participants with complete spirometry data and at least five wheeze assessments (n=1964), (bottom) participants with complete data on spirometry and at least ten wheeze assessments (n=1737).

Each panel presents a trajectory group illustrating the parallel evolution of wheeze prevalence (%) (red left y-axis) and mean FEV₁/FVC GLI percent predicted (blue, right y-axis). Trajectories were derived by joint modelling of wheeze and lung function (FEV₁/FVC), i.e., both features were modelled together to capture the developmental patterns of symptoms and lung function.

NIFW: never/infrequent wheeze; ETW: early transient wheeze; LOW: late onset wheeze; PEW: persistent wheeze; NLF: normal lung function; RLF: reduced lung function

| 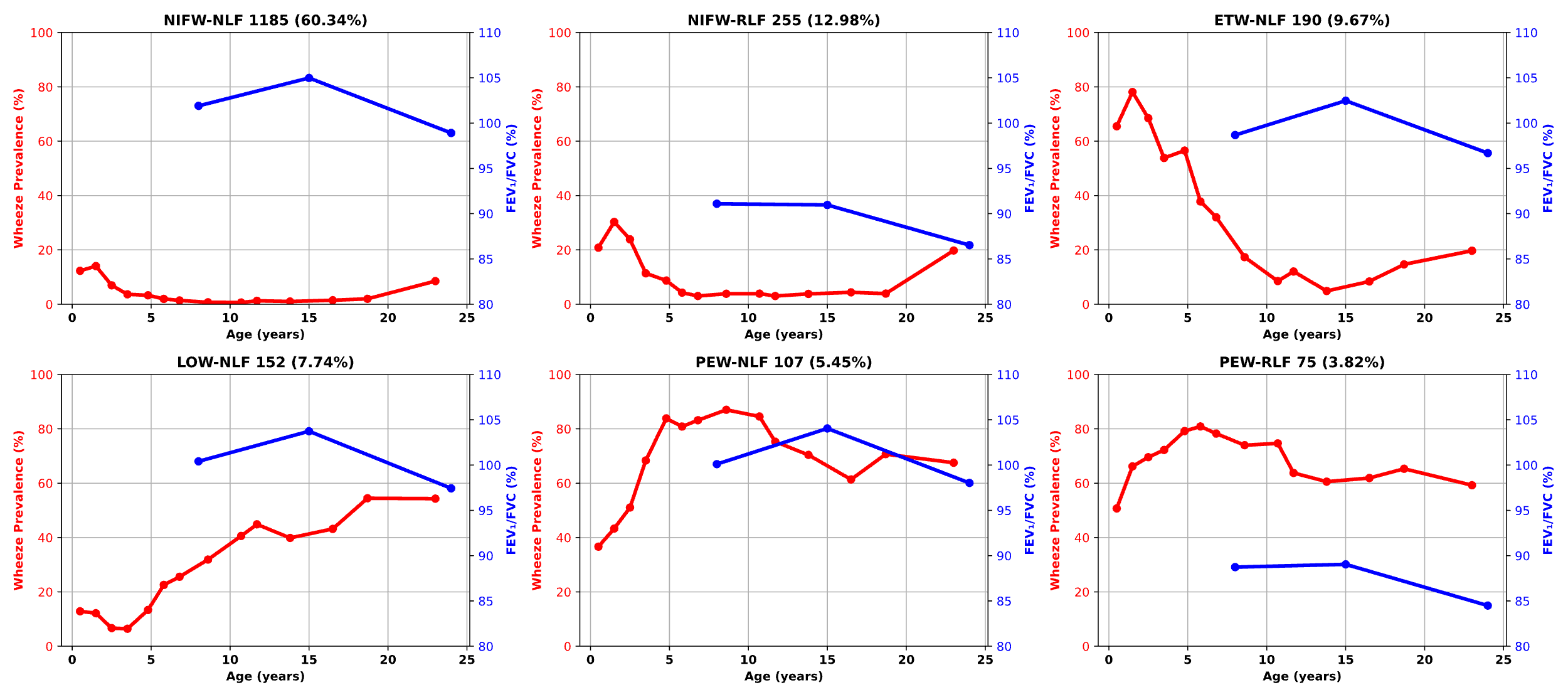 |
| --- |
| 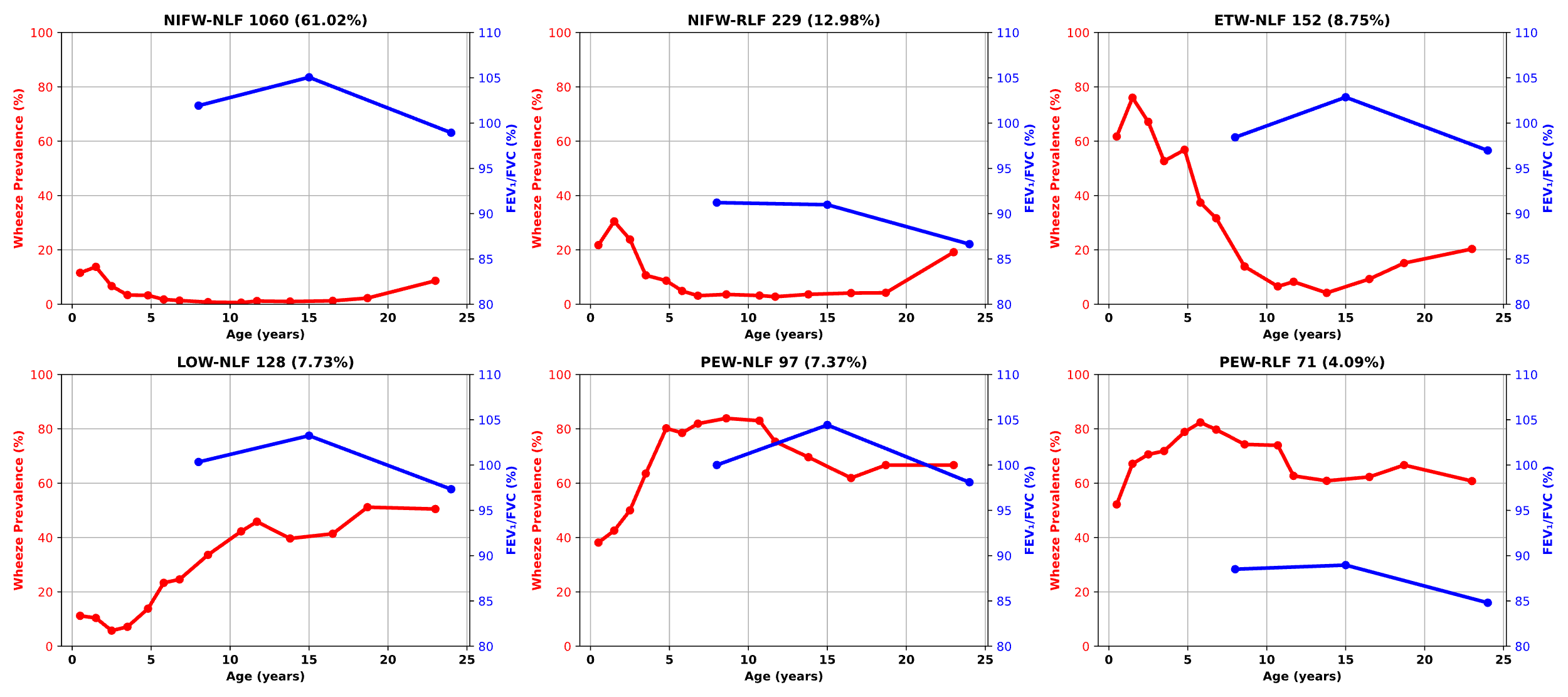 |

**Table S7:** Confusion matrix showing similarity in trajectory allocation when using participants with at least two spirometry and three wheeze assessments, and trajectory allocation when using participants with complete spirometry data and at least five wheeze assessments. This shows a high similarity between analyses of partially complete and incomplete data. Data from the discovery cohort (n=1964).

NIFW: never/infrequent wheeze; ETW: early transient wheeze; LOW: late onset wheeze; PEW: persistent wheeze; NLF: normal lung function; RLF: reduced lung function

|  |  | Trajectories based on complete spirometry data and at least five wheeze assessments | | | | | |
| --- | --- | --- | --- | --- | --- | --- | --- |
|  |  | NIFW-NLF | NIFW-RLF | ETW-NLF | LOW-NLF | PEW-NLF | PEW-RLF |
| Trajectories based on at least two spirometry and three wheeze assessments | NIFW-NLF | **1182 (96.96%)** | 0 | 3 (1.48%) | 0 | 0 | 0 |
|  | NIFW-RLF | 20 (1.64%) | **233 (100%)** | 0 | 2 (1.31%) | 0 | 0 |
|  | ETW-NLF | 1 (0.08%) | 0 | **189 (93.10%)** | 0 | 0 | 0 |
|  | LOW-NLF | 16 (1.31%) | 0 | 1(0.49%) | **135 (88.23%)** | 0 | 0 |
|  | PEW-NLF | 0 | 0 | 8 (3.94%) | 14 (9.15%) | **85 (97.71%)** | 0 |
|  | PEW-RLF | 0 | 0 | 2 (0.98%) | 2 (0.31%) | 2 (2.29%) | **69 (100%)** |

**Table S8:** Confusion matrix showing similarity in trajectory allocation when using participants with at least two spirometry and three wheeze assessments, and trajectory allocation when using participants with complete spirometry data and at least 10 wheeze assessments. This shows a high similarity between analyses of partially complete and incomplete data. Data from the discovery cohort (n=1737).

NIFW: never/infrequent wheeze; ETW: early transient wheeze; LOW: late onset wheeze; PEW: persistent wheeze; NLF: normal lung function; RLF: reduced lung function

|  |  | Trajectories based on complete spirometry data and at least 10 wheeze assessments | | | | | |
| --- | --- | --- | --- | --- | --- | --- | --- |
|  |  | NIFW-NLF | NIFW-RLF | ETW-NLF | LOW-NLF | PEW-NLF | PEW-RLF |
| Trajectories based on at least two spirometry and three wheeze assessments | NIFW-NLF | **1057 (99.72%)** | 20 (8.73%) | 3 (1.97%) | 11 (8.59%) | 0 | 0 |
|  | NIFW-RLF | 0 | **207 (90.39%)** | 1 (0.66%) | 0 | 0 | 0 |
|  | ETW-NLF | 3 (0.28%) | 0 | **147 (96.71%)** | 1 (0.78%) | 9 (9.28%) | 2 (2.82%) |
|  | LOW-NLF | 0 | 2 (0.87%) | 1 (0.66%) | **116 (90.62%)** | 16 (16.49% | 2 (2.82%) |
|  | PEW-NLF | 0 | 0 | 0 | **0** | **72 (74.23%)** | 3 (4.22%) |
|  | PEW-RLF | 0 | 0 | 0 | 0 | 0 | **64 (90.14%)** |

**Figure S7:** Wheeze severity and cough with doctor visit by trajectories in the discovery population:

Panel A: Wheeze frequency, Panel B: Wheeze disturbs speech, Panel C: Wheeze limits sleep, and Panel D: cough with doctor visit.

Wheeze frequency: 3 or more wheeze episodes in the last 12 months.

NIFW: never/infrequent wheeze; ETW: early transient wheeze; LOW: late onset wheeze; PEW: persistent wheeze; NLF: normal lung function; RLF: reduced lung function


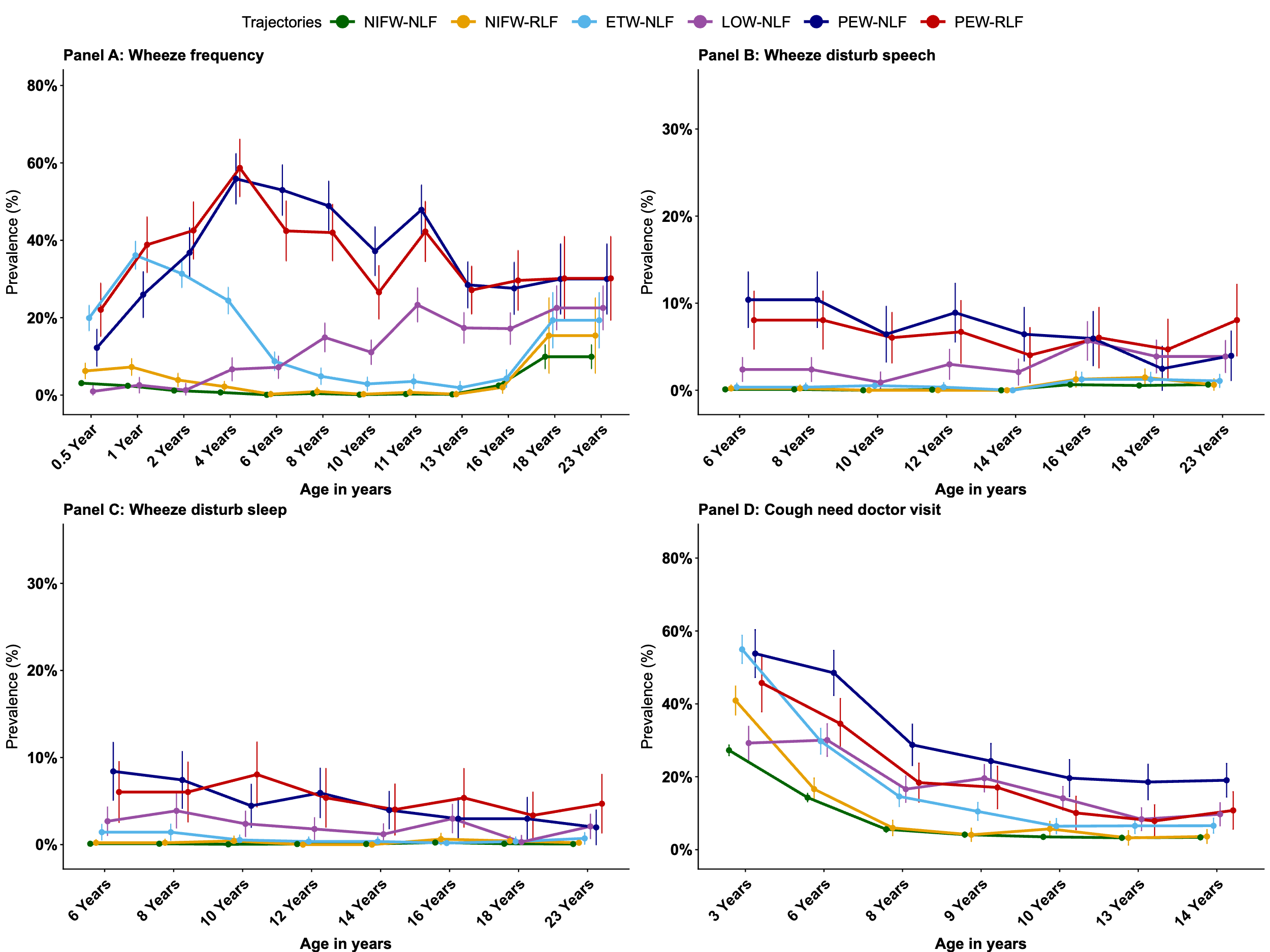


**Figure S8:** Boxplots of exhaled FeNO (n=1761), FEV₁ reversibility (n=3225), total serum IgE (n=2857), and methacholine dose response slope (DRS) (n=2873) across trajectory groups.

IgE, DRS, and FeNO were natural log-transformed; FEV₁ reversibility was transformed using a signed log function to accommodate negative values. The letter display indicates pairwise comparisons: groups sharing the same letter are not significantly different.

NIFW: never/infrequent wheeze; ETW: early transient wheeze; LOW: late onset wheeze; PEW: persistent wheeze; NLF: normal lung function; RLF: reduced lung function

**
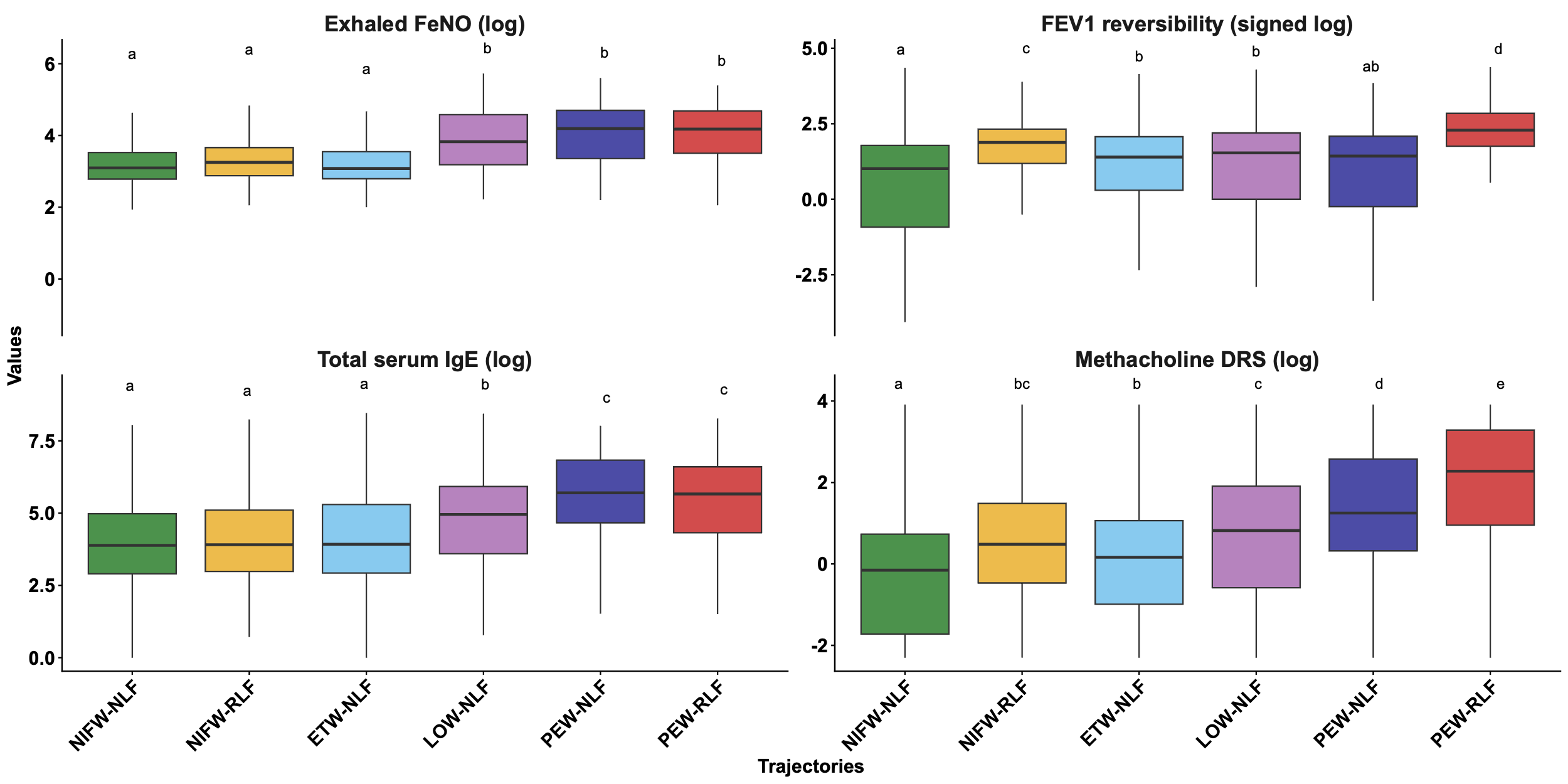
**

**
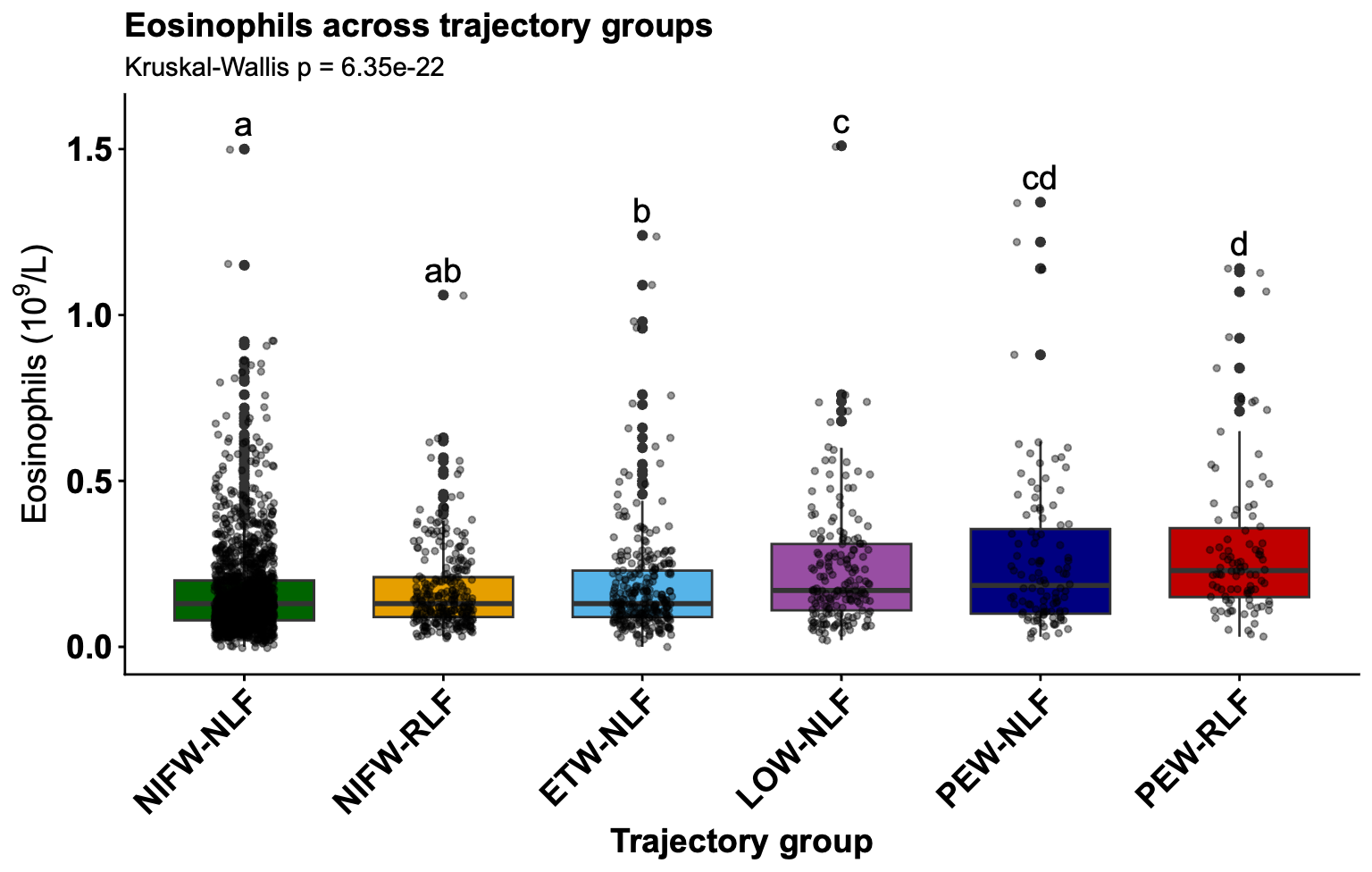
**Figure S9: Box plots show median and interquartile range of blood eosinophils across trajectory groups. Groups sharing the same letter are not significantly different (pairwise Wilcoxon tests with BH correction).

**Table S9:** Early life and parental risk factors associated with trajectories; NIFW-NL as a reference. Data are adjusted relative risk ratio (Wald 95% CI) from the discovery population (ALSPAC) and Wald χ^2^ p values. For birth weight, the relative risk ratio is per one kilogram increase; for financial difficulty score, anxiety score, and depression score, the relative risk ratios are per a five-unit increase. For categorical variables, the relative risk ratios are for “yes”, with “no” as a reference. RRR: relative risk ratio.

RRRs for each variable are estimated from models adjusted for sex, prematurity, maternal asthma, and maternal smoking. For variables among the adjustment set (e.g., sex), the RRR represents the association adjusted for the remaining covariates in the set.

Financial difficulties score at 32 weeks of gestation. Anxiety and depression scores at 18 weeks of gestation. Breastfed ever: by 15 months of age

NIFW: never/infrequent wheeze; ETW: early transient wheeze; LOW: late onset wheeze; PEW: persistent wheeze; NLF: normal lung function; RLF: reduced lung function

|  | ETW-NLF | | NIFW-RLF | | LOW-NLF | | PEW-NLF | | PEW-RLF | |
| --- | --- | --- | --- | --- | --- | --- | --- | --- | --- | --- |
| Covariates | Adjusted RRR and 95% CI | P-value | Adjusted RRR and 95% CI | P-value | Adjusted RRR and 95% CI | P-value | Adjusted RRR and 95% CI | P-value | Adjusted RRR and 95% CI | P-value |
| Male | 1.48 (1.23,1.79) | **<.001** | 1.02 (0.83,1.24) | 0.877 | 1.21 (0.96,1.52) | 0.115 | 1.75 (1.30,2.36) | **<.001** | 1.70 (1.20,2.40) | **0.003** |
| Prematurity | 1.87 (1.28,2.74) | **0.001** | 1.24 (0.78,1.96) | 0.368 | 0.59 (0.29,1.22) | 0.157 | 0.76 (0.33,1.76) | 0.521 | 2.12 (1.13,3.99) | **0.020** |
| Maternal smoking | 1.42 (1.13,1.78) | **0.003** | 1.61 (1.27,2.04) | **<.001** | 1.37 (1.03,1.83) | **0.029** | 1.04 (0.70,1.54) | 0.845 | 1.90 (1.29,2.80) | **0.001** |
| Maternal asthma pregnancy | 2.38 (1.84,3.08) | **<.001** | 1.06 (0.75,1.50) | 0.745 | 1.88 (1.35,2.62) | **<.001** | 3.13 (2.18,4.51) | **<.001** | 4.02 (2.70,5.99) | **<.001** |
| Birth Weight in kgs | 0.98 (0.80,1.20) | 0.851 | 0.96 (0.77,1.18) | 0.682 | 0.85 (0.66,1.09) | 0.198 | 1.15 (0.84,1.57) | 0.386 | 0.59 (0.41,0.84) | **0.004** |
| Maternal hayfever pregnancy | 1.42 (1.15,1.75) | **0.001** | 0.99 (0.78,1.26) | 0.937 | 1.59 (1.23,2.05) | **<.001** | 1.43 (1.03,1.98) | **0.033** | 1.17 (0.79,1.74) | 0.422 |
| Maternal eczema pregnancy | 1.23 (0.99,1.52) | 0.058 | 0.95 (0.74,1.21) | 0.677 | 1.13 (0.86,1.48) | 0.377 | 1.61 (1.17,2.21) | **0.003** | 1.61 (1.11,2.34) | **0.012** |
| Delivery-Normal | 1.15 (0.92,1.43) | 0.235 | 0.94 (0.75,1.19) | 0.616 | 0.84 (0.65,1.10) | 0.208 | 0.83 (0.60,1.16) | 0.278 | 0.89 (0.60,1.31) | 0.553 |
| Breastfed ever | 0.72 (0.56,0.91) | **0.007** | 0.96 (0.73,1.26) | 0.765 | 1.13 (0.81,1.58) | 0.471 | 0.93 (0.62,1.40) | 0.729 | 1.14 (0.70,1.87) | 0.596 |
| Financial Difficulties score | 1.25 (1.08,1.44) | **0.003** | 1.09 (0.93,1.28) | 0.292 | 1.19 (0.99,1.42) | 0.063 | 1.29 (1.03,1.62) | **0.025** | 1.17 (0.89,1.54) | 0.255 |
| EPD Score | 1.25 (1.13,1.39) | **<.001** | 1.12 (1.00,1.25) | 0.060 | 1.05 (0.92,1.21) | 0.469 | 1.32 (1.12,1.55) | **<.001** | 0.98 (0.80,1.21) | 0.880 |
| Crown Crisp-Anxiety Score | 1.36 (1.18,1.56) | **<.001** | 1.05 (0.90,1.24) | 0.521 | 1.06 (0.88,1.27) | 0.557 | 1.44 (1.16,1.79) | **0.001** | 1.09 (0.83,1.44) | 0.534 |
| Crown Crisp-Depression Score | 1.26 (1.07,1.48) | **0.006** | 1.07 (0.89,1.28) | 0.453 | 1.06 (0.86,1.31) | 0.578 | 1.35 (1.05,1.74) | **0.020** | 0.87 (0.62,1.21) | 0.401 |

**Figure S10: Heatmaps of maternal smoking and high environmental tobacco smoke (ETS) exposure across six joint trajectories of** current **wheeze and** FEV₁/FVC**.**

Panel A shows the prevalence of maternal smoking at four critical periods: ever smoked, pre-pregnancy, first trimester, and the last two weeks of pregnancy. Panel B illustrates the prevalence of ETS exposure from infancy through early childhood (0.5 to 5.5 years). Each cell represents the percentage of individuals in each wheeze trajectory group with the corresponding exposure. Higher exposure: exposed for more than 3 hours a week.

NIFW: never/infrequent wheeze; ETW: early transient wheeze; LOW: late onset wheeze; PEW: persistent wheeze; NLF: normal lung function; RLF: reduced lung function


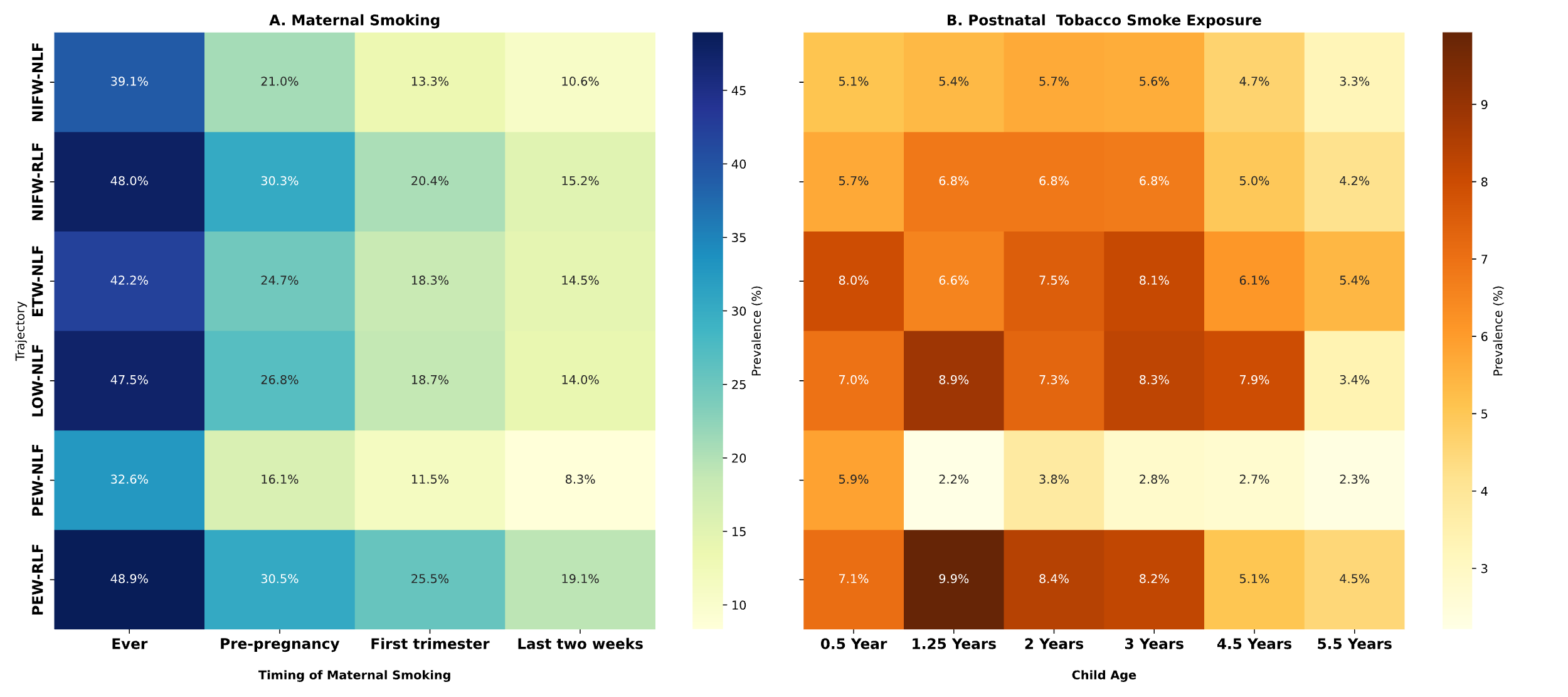


**Figure S11:** Active smoking **across six joint trajectories of wheeze and FEV_1_/FVC.**

Panel A: Prevalence of active smoking with 95% CIs from adolescence to early adulthood, by joint wheeze and FEV₁/FVC trajectories. Bars represent prevalence for smoking, and error bars indicate 95% CIs.

Panel B: Relative risk ratios with 95% CIs for active smoking from adolescence to early adulthood. Data are unadjusted relative risk ratios (Wald 95% CI) from the discovery population (ALSPAC). NIFW-NLF is used as a reference. At each age, the relative risk ratios are for “yes”, with “no” as a reference. RRR: relative risk ratio.

NIFW: never/infrequent wheeze; ETW: early transient wheeze; LOW: late onset wheeze; PEW: persistent wheeze; NLF: normal lung function; RLF: reduced lung function


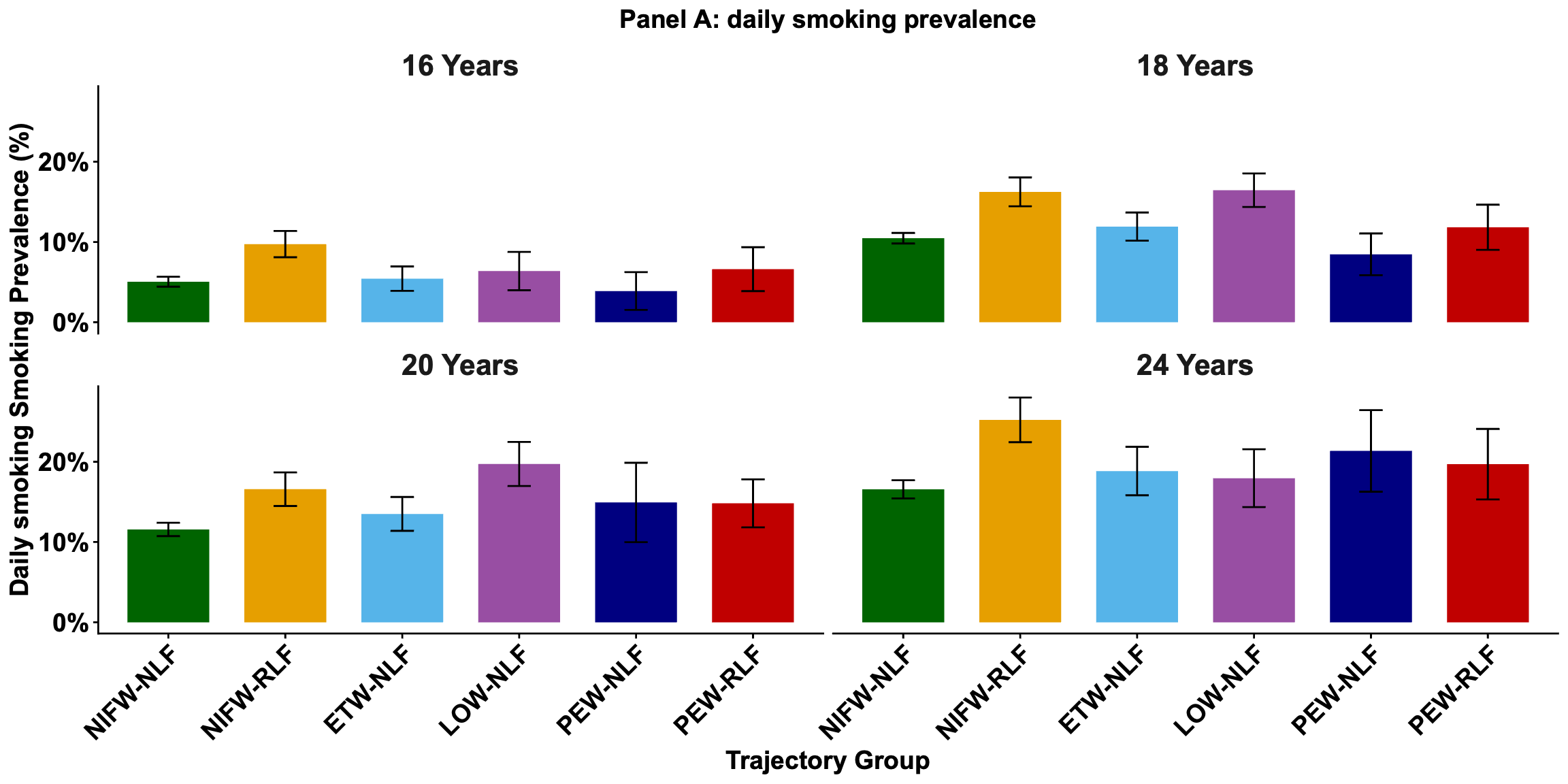


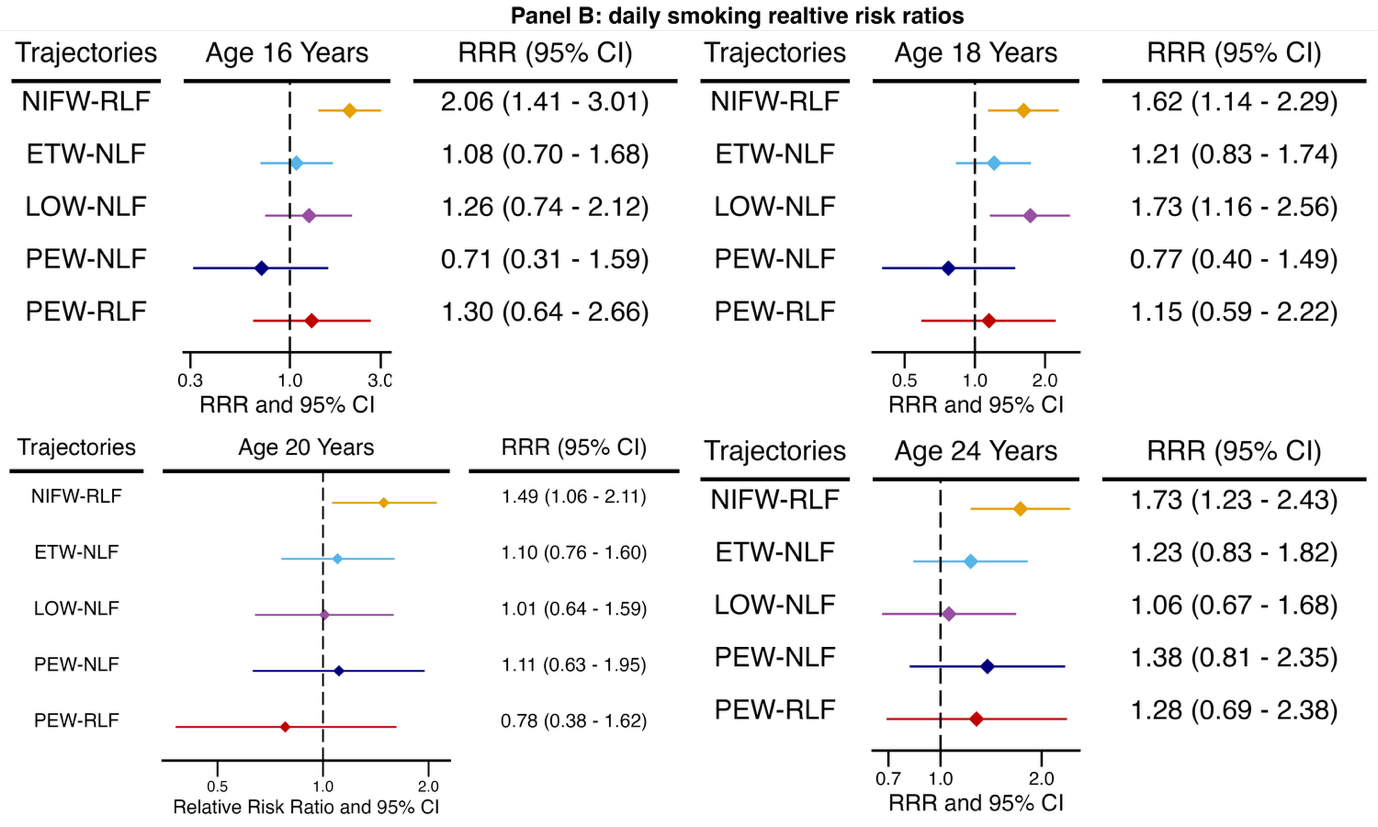


**Figure S12**. Association of body fat mass in adolescence (age 10, 12, 14, and 15 years) with trajectories. Data are unadjusted relative risk ratios (Wald 95% CI) from the discovery population (ALSPAC). NIFW-NLF is used as a reference. At each age, the relative risk ratio is for a one-unit increase in body fat mass.

NIFW: never/infrequent wheeze; ETW: early transient wheeze; LOW: late onset wheeze; PEW: persistent wheeze; NLF: normal lung function; RLF: reduced lung function

**
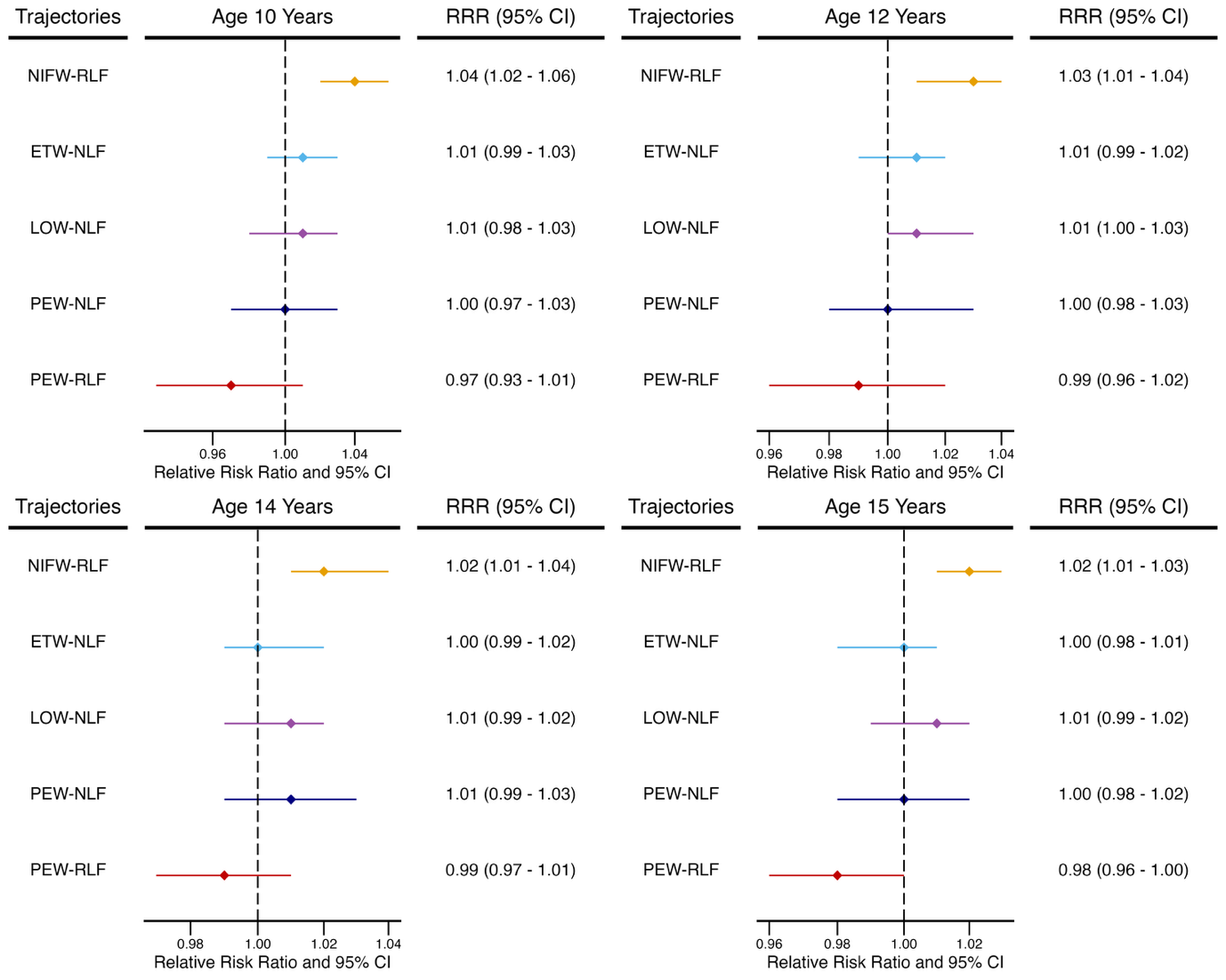
**

# **REPLICATION POPULATIONS**

## ***Final Model selection first replication populations (pooled data from MAAS and IOW):***

We began by fitting a two-trajectory model, assuming only two underlying groups. We then fit additional models with an increasing number of trajectories up to 20. The FCAP plot is given in Figures S11 and S12. For the MAAS and IOW pooled data, for BIC, we did not experience the asymptotic behaviour expected for large sample sizes, as the sample was smaller compared to the discovery population. The highest BIC was observed for the 6-class model. 6-class model also satisfies other criteria, i.e., the minimum average posterior probability was greater than 70%, and the smallest class size was more than 1%. Thus, the final selected model for the replication cohorts was a 6-class multi-trajectory model.

**Figure S13:** Comparison of model fit and classification metrics across trajectory solutions ranging from 2 to 20 classes. The main plot displays the BIC, while secondary axes represent average posterior probability (AvePP), mismatch rate, and the standard deviation (SD) of posterior probabilities. ΔBIC values between successive models are annotated. The 6-class model (highlighted in yellow) was selected based on its optimal trade-off between statistical fit and classification accuracy, with minimal additional benefit observed in more complex models.

**AvePP:** Average posterior probability; **AvePP -:** minimum average posterior probability across classes, **Mismatch +:** maximum mismatch across classes; **SD+:** maximum standard deviation of posterior probability across classes. ΔBIC: difference between successive BIC values.

**
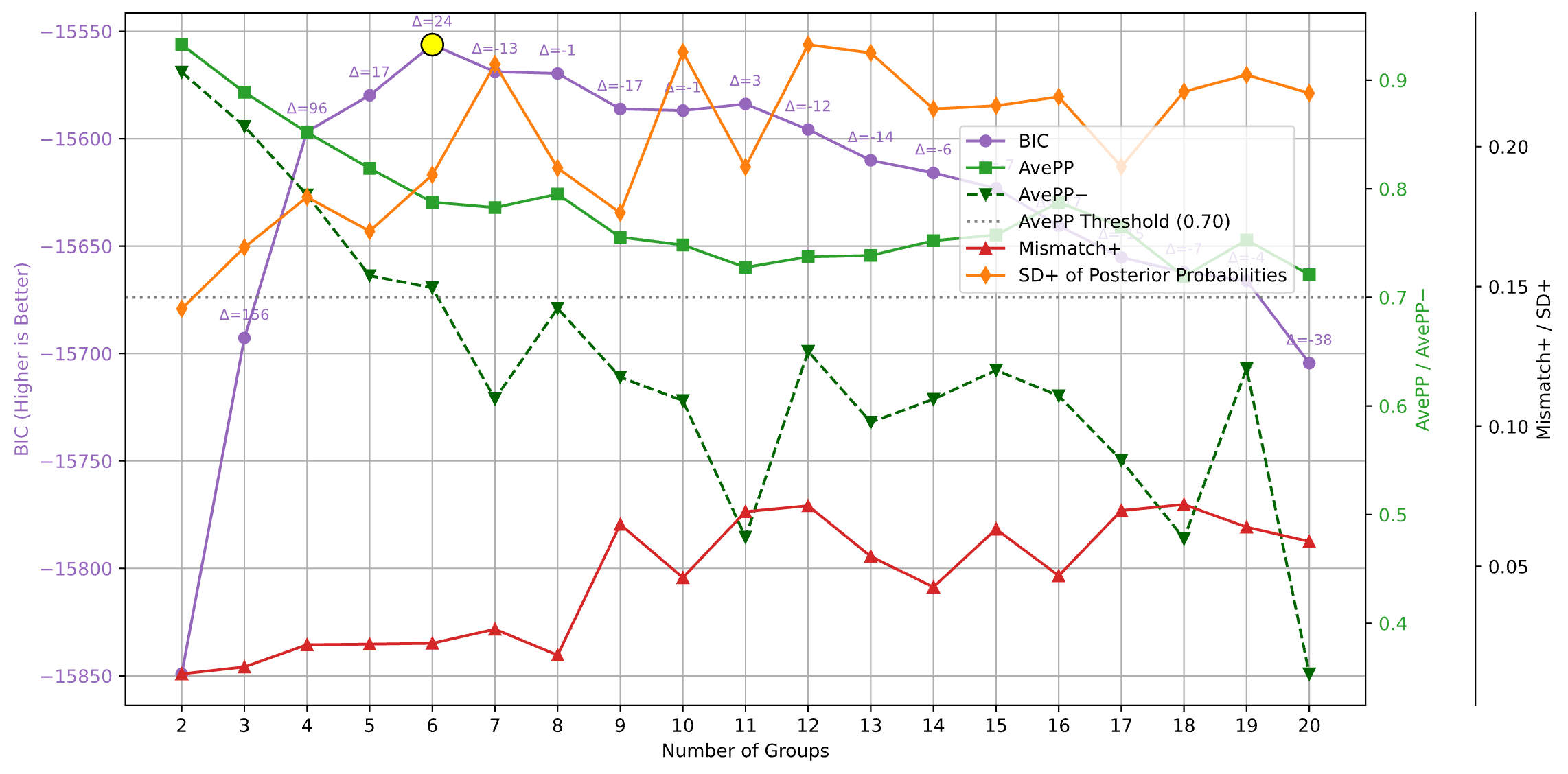
**

**Figure S14:** Fit-Criteria Assessment Plots for MAAS replication population.

1. Akaike's information criterion, Bayesian information criterion and Likelihood.
2. Posterior probability, mismatch and SD
3. Odds of correct classification
4. Percentage of individuals belonging to the smallest group

A)

B)

C)

D)

## ***Final Model selection second replication populations (BAMSE):***

We began by fitting a two-trajectory model, assuming only two underlying groups. We then fit additional models with an increasing number of trajectories up to 15. The FCAP plot is given in Figure S13. For the BAMSE data, the BIC exhibits the asymptotic behaviour expected for large sample sizes, as the sample was larger compared to the first replication populations. The improvement in BIC after 6 classes were lower; furthermore, the average lowest posterior probabilities after 6 classes were lower than the threshold of 0.7. Thus, the final selected model for the second replication population was a 6-class multi-trajectory model.

**Figure S15:** Comparison of model fit and classification metrics across trajectory solutions ranging from 2 to 15 classes. The main plot displays the BIC, while secondary axes represent average posterior probability (AvePP), mismatch rate, and the standard deviation (SD) of posterior probabilities. ΔBIC values between successive models are annotated. The 6-class model (highlighted in yellow) was selected based on its optimal trade-off between statistical fit and classification accuracy, with minimal additional benefit observed in more complex models.

**AvePP:** Average posterior probability; **AvePP -:** minimum average posterior probability across classes, **Mismatch +:** maximum mismatch across classes; **SD+:** maximum standard deviation of posterior probability across classes. ΔBIC: difference between successive BIC values.

**
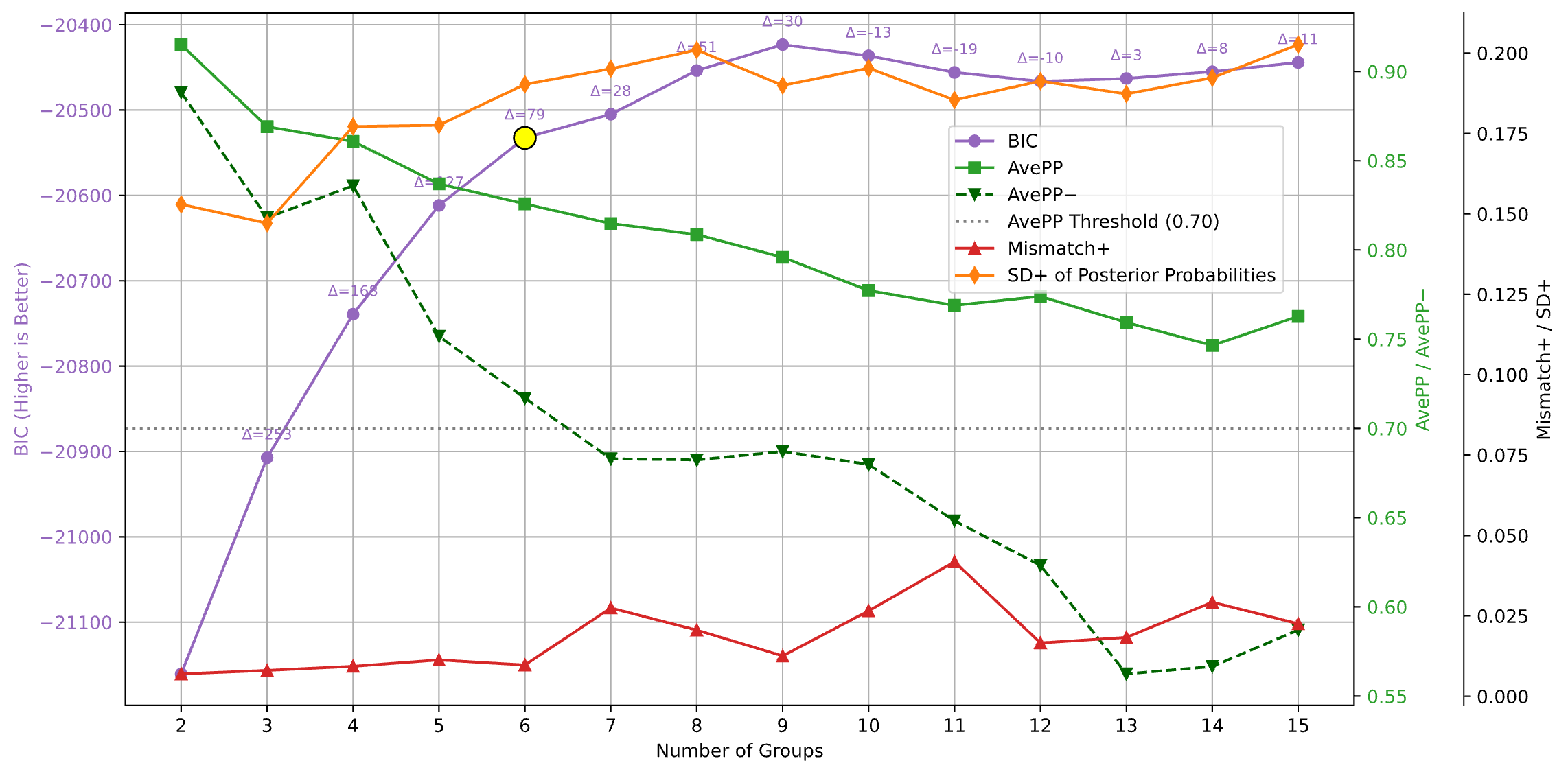
**

## ***Trajectories from first replication populations (pooled data from MAAS and IOW):***

**Figure S16:** Change in the current **wheeze prevalence and** mean FEV₁/FVC GLI percent predicted values **with age, by wheeze and** FEV₁/FVC joint trajectories**, using data from the first replication population (n=1378).**

Each panel presents a trajectory group illustrating the parallel evolution of wheeze prevalence (%) (red left y-axis) and mean FEV₁/FVC GLI percent predicted (blue, right y-axis). Trajectories were derived by joint modelling of wheeze and lung function (FEV₁/FVC), i.e., both features were modelled together to capture the developmental patterns of symptoms and lung function.

NIWZ = never/infrequent wheeze; ETW: early transient wheeze; LOW = late onset wheeze; PEW: persistent wheeze; NLF: normal lung function; RLF: reduced lung function.


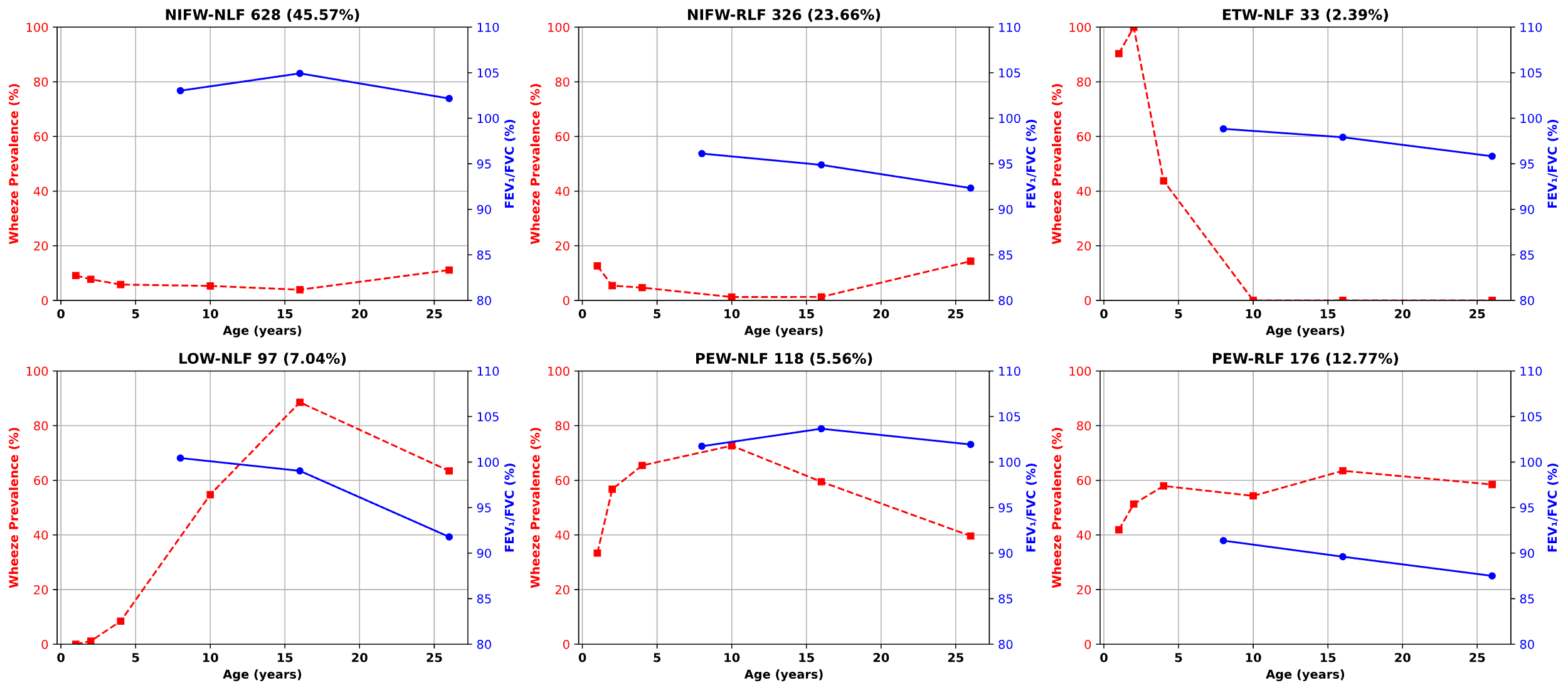


## ***Trajectories from the second replication populations (BAMSE):***

**Figure S17:** Change in the current **wheeze prevalence and** mean FEV₁/FVC GLI percent predicted values **with age, by wheeze and** FEV₁/FVC joint trajectories**, using data from the second replication population (n=2010).**

Each panel presents a trajectory group illustrating the parallel evolution of wheeze prevalence (%) (red left y-axis) and mean FEV₁/FVC GLI percent predicted (blue, right y-axis). Trajectories were derived by joint modelling of wheeze and lung function (FEV₁/FVC), i.e., both features were modelled together to capture the developmental patterns of symptoms and lung function.

NIWZ = never/infrequent wheeze; ETW: early transient wheeze; LOW = late onset wheeze; PEW: persistent wheeze; NLF: normal lung function; RLF: reduced lung function.

**
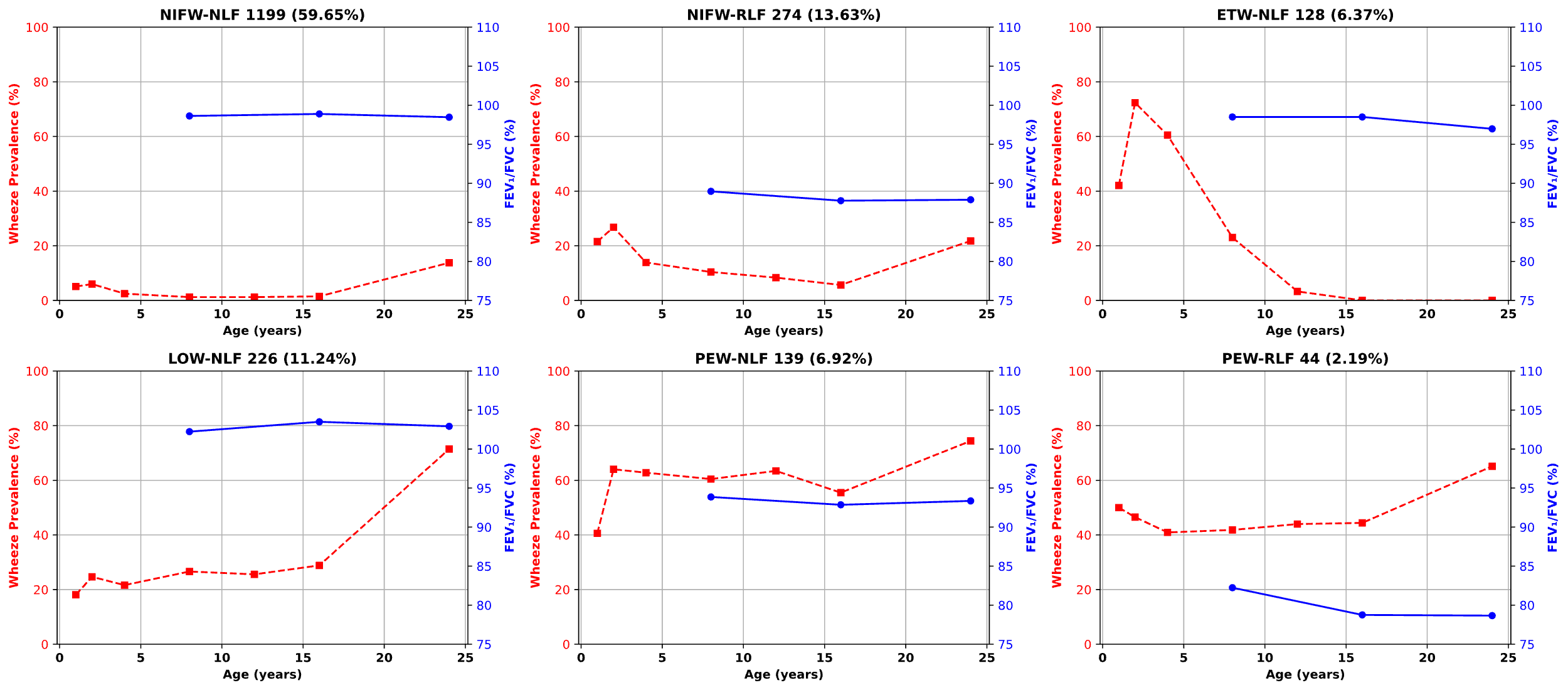
**

## ***Selected association analysis from both replication populations***

**Table S10:** Associations of trajectories with FeNO in all replication cohorts.

NIWZ = never/infrequent wheeze; ETW: early transient wheeze; LOW = late onset wheeze; PEW: persistent wheeze; NLF: normal lung function; RLF: reduced lung function.

|  | **FeNO at 11 years**  **MAAS** | **FeNO at 16 years**  **MAAS** | **FeNO at 20 years**  **MAAS** | **FeNO at 26 years**  **IOW** | **FeNO at 16 years**  **BAMSE** | **FeNO at 24 years**  **BAMSE** |
| --- | --- | --- | --- | --- | --- | --- |
| Trajectories | Median (Q1-Q3) | Median (Q1-Q3) | Median (Q1-Q3) | Median (Q1-Q3) | Median (Q1-Q3) | Median (Q1-Q3) |
| NIFW-NLF | 9.5 (7.10-13.0) | 15.0 (10.0-26.0) | 13.00 (8.0-21.0) | 14.0 (10.0-20.5) | 15.0 (11.0-21.0) | 11.0 (8.0-17.0) |
| NIFW-RLF | 9.3 (7.10-14.5) | 17.0 (12.0-28.0) | 12.00 (7.0-19.0) | 14.0 (9.0-18.0) | 15.0 (11.0-22.0) | 12.0 (9.0-18.0) |
| ETW-NLF | 7.4 (5.60-18.5) | 17.5 (11.0-37.5) | 9.50 (5.0-19.0) | 11.0 (9.5-28.5) | 14.0 (10.0-19.0) | 12.0 (8.0-17.0) |
| LOW-NLF | 10.9 (8.80-25.5) | 26.3 (14.0-44.0) | 15.50 (8.0-55.0) | 19.5 (12.0-37.0) | 18.0 (12.0-28.0) | 13.0 (9.0-23.0) |
| PEW-NLF | 24.4 (9.3-56.3) | 26.0 (15.4-53.0) | 18.00 (9.0-44.0) | 16.0 (10.0-25.0) | 22.0 (13.0-41.0) | 13.0 (9.0-26.0) |
| PEW-RLF | 20.8 (8.0-50.0) | 25.0 (14.0-79.9) | 19.00 (8.0-37.0) | 19.5 (10.0-33.5) | 21.0 (12.0-31.0) | 13.0 (8.0-23.0) |

**Table S11:** Associations of trajectories BDR in all replication cohorts.

NIWZ = never/infrequent wheeze; ETW: early transient wheeze; LOW = late onset wheeze; PEW: persistent wheeze; NLF: normal lung function; RLF: reduced lung function.

| Trajectories | **BDR >12%**  **at 5 years**  **MAAS** | **BDR >12%**  **at 11 years**  **MAAS** | **BDR >12%**  **at 16 years**  **MAAS** | **BDR >12%**  **at 18 years**  **IOW** | **BDR >12%**  **at 26 years**  **IOW** | **BDR > 12%**  **at 24 years**  **BAMSE** |
| --- | --- | --- | --- | --- | --- | --- |
|  | n/N (%) | n/N (%) | n/N (%) | n/N (%) | n/N (%) | n/N (%) |
| NIFW-NLF | 11/229 (4.80%) | 12/259 (4.63%) | 6/256 (2.34%) | 5/320 (1.56%) | 0/202 (0.00%) | 1/910 (~0%) |
| NIFW-RLF | 9/104 (8.65%) | 10/119 (8.40%) | 13/117 (11.11%) | 9/170 (5.29%) | 5/137 (3.65%) | 7/226 (0.03%) |
| ETW-NLF | 2/17 (11.76%) | 3/21 (14.29%) | 2/19 (10.53%) | 0/9 (0.00%) | 0/4 (0%) | 0/93 (0%) |
| LOW-NLF | 2/10 (20.00%) | 5/14 (35.71%) | 0/14 (0.00%) | 4/68 (5.88%) | 5/56 (8.93%) | 0/184 (0%) |
| PEW-NLF | 6/45 (13.33%) | 8/57 (14.04%) | 2/56 (3.57%) | 2/46 (4.35%) | 0/32 (0%) | 1/104 (~0%) |
| PEW-RLF | 17/55 (30.91%) | 24/73 (32.88%) | 16/69 (23.19%) | 26/90 (28.89%) | 11/68 (16.18%) | 6/31 (19.35%) |

**Table S12:** Prevalence of airway hyperreactivity (AHR) to methacholine at the maximum administered dose of 16 mg/mL, stratified by trajectory group in MAAS and IOW. Values shown are the number of participants with valid test results, the number meeting the AHR criterion, and the corresponding prevalence (%).

NIWZ = never/infrequent wheeze; ETW: early transient wheeze; LOW = late onset wheeze; PEW: persistent wheeze; NLF: normal lung function; RLF: reduced lung function.

|  | IOW | | MAAS | | |
| --- | --- | --- | --- | --- | --- |
| Trajectories | Age 10 | Age 18 | Age 8 | Age 11 | Age 20 |
| NIFW-NLF | 59/235 (25.1%) | 6/238 (2.5%) | 20/192 (10.4%) | 50/204 (24.5%) | 8/194 (4.12%) |
| NIFW-RLF | 51/137 (37.2%) | 10/128 (7.8%) | 10/95 (10.5%) | 26/97 (26.8%) | 11/84 (13.1%) |
| ETW-NLF | 5/11 (45.5%) | 0/6 (0.0%) | 4/17 (23.5%) | 7/16 (43.8%) | 1/11 (9.09%) |
| LOW-NLF | 41/69 (59.4%) | 14/56 (25.0%) | 2/12 (16.7%) | 10/12 (83.3%) | 2/6 (33.3%) |
| PEW-NLF | 23/51 (45.1%) | 6/34 (17.6%) | 14/42 (33.3%) | 20/45 (44.4%) | 9/42 (21.4%) |
| PEW-RLF | 57/87 (65.5%) | 18/66 (27.3) | 29/64 (45.3%) | 33/57 (57.9%) | 17/50 (34.0%) |

**Figure S18:** The proportion of children with allergic sensitisation across trajectories.

For the MAAS and IOW skin prick test, sensitisation was defined as one or more positive skin prick tests with a 3mm cut-off to the most common allergens. For BAMSE IgE sensitisation was defined as airborne allergen-specific IgE ≥0·35 kUA/L.

NIWZ = never/infrequent wheeze; ETW: early transient wheeze; IETW-RLF: infrequent early transient wheeze; LOW = late onset wheeze; PEW: persistent wheeze; NLF: normal lung function; RLF: reduced lung function.


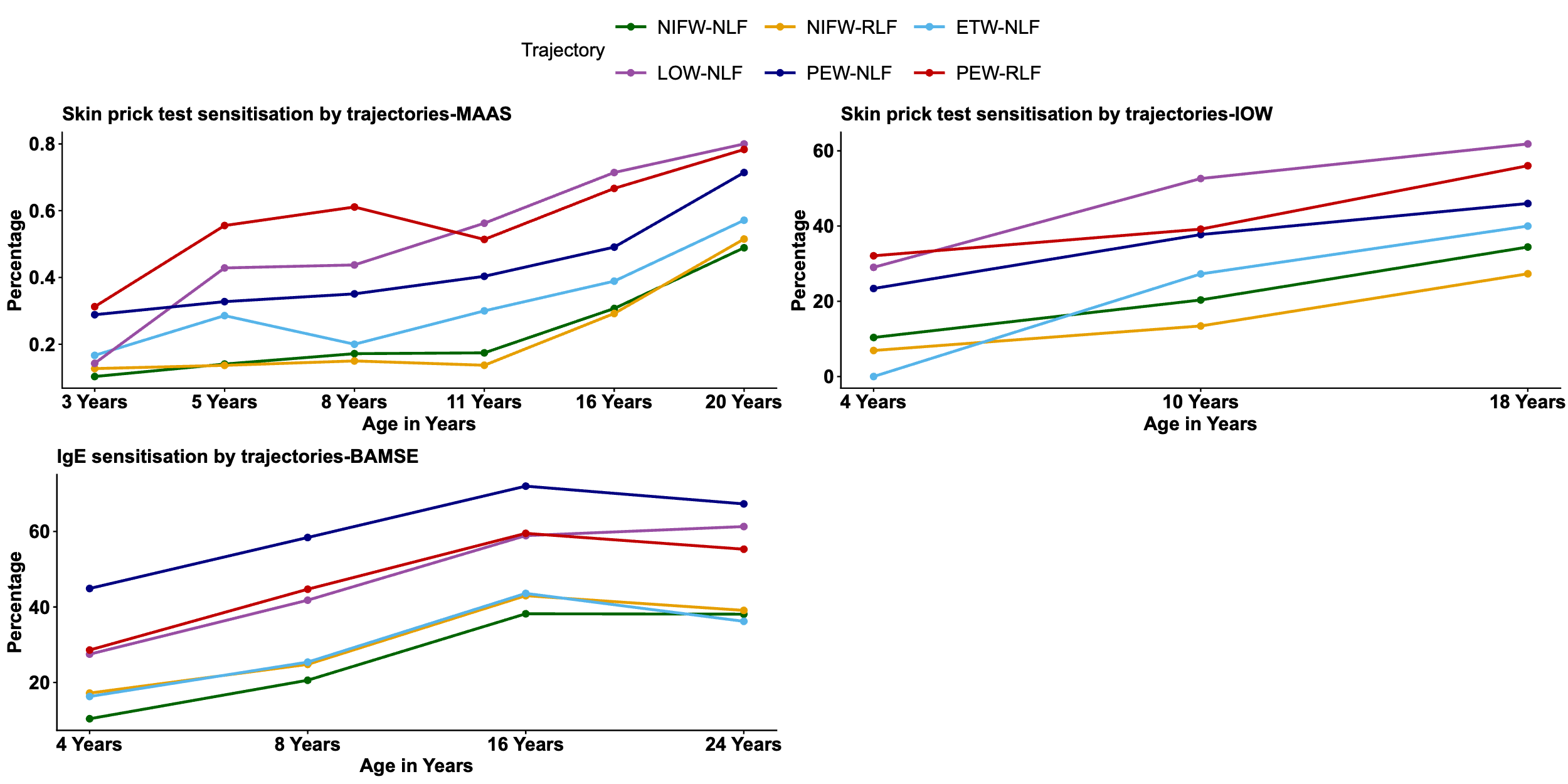


**Figure S19:** The proportion of children with severe asthma or wheeze that required hospital admission or oral steroids across trajectories (MAAS only).

**
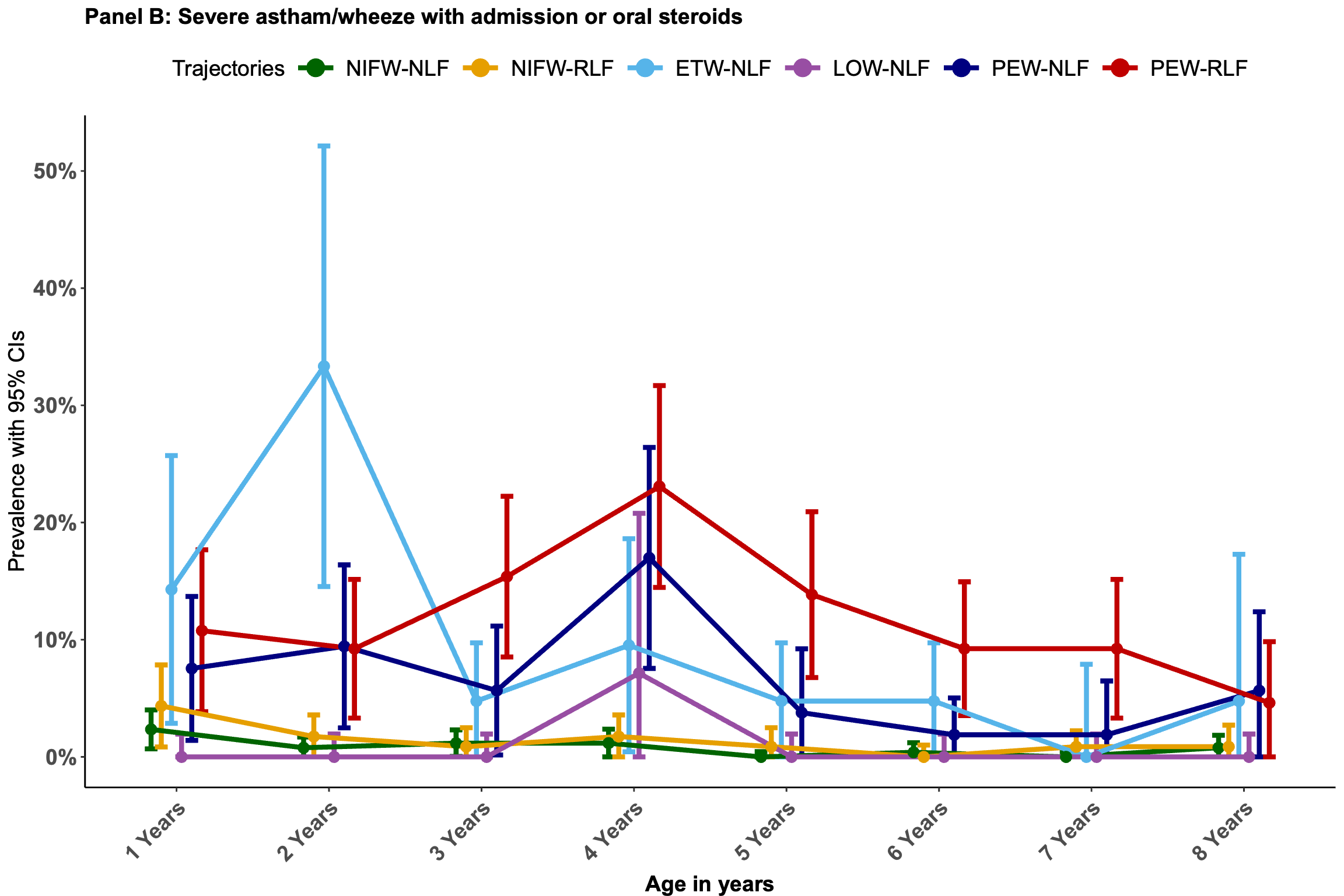
**NIWZ = never/infrequent wheeze; ETW: early transient wheeze; LOW = late onset wheeze; PEW: persistent wheeze; NLF: normal lung function; RLF: reduced lung function.

**Table S13:** Early life and parental risk factors associated with trajectories in first replication analysis; NIFW-NL as a reference. Data are relative risk ratios (Wald 95% CI) from the replication cohorts and Wald χ^2^ p values. For all continuous variables, the relative risk ratios are per unit increase, whereas for categorical variables, the relative risk ratios are for “yes”, with “no” as a reference. RRR: relative risk ratio. Empty cells represent cells with no or limited cases.

|  | **NIFW-RLF** | | **ETW-NLF** | | **LOW NLF** | | **PEW NLF** | | **PEW RLF** | |
| --- | --- | --- | --- | --- | --- | --- | --- | --- | --- | --- |
| **Risk Factors** | **Univariate RRR (95% CI)** | **P-value** | **Univariate RRR (95% CI)** | **P-value** | **Univariate RRR (95% CI)** | **P-value** | **Univariate RRR (95% CI)** | **P-value** | **Univariate**  **RRR (95% CI)** | **P-value** |
| Male | 0.76 (0.59,0.99) | 0.040 | 1.26 (0.63,2.51) | 0.519 | 0.72 (0.31,1.68) | 0.448 | 1.17 (0.56,2.44) | 0.671 | 1.13 (0.83,1.55) | 0.434 |
| Gestational age in weeks | 0.98 (0.90,1.07) | 0.614 | 0.90 (0.72,1.12) | 0.350 | 1.05 (0.77,1.42) | 0.765 | 0.87 (0.70,1.08) | 0.209 | 0.89 (0.80,0.98) | 0.016 |
| Prematurity | 1.30 (0.57,2.97) | 0.536 | 4.58 (1.14,18.29) | 0.031 |  |  | 1.62 (0.19,13.78) | 0.658 | 3.98 (1.90,8.36) | <.001 |
| Birth weight in kg | 1.00 (1.00,1.00) | 0.964 | 0.99 (0.98,1.01) | 0.401 | 1.00 (0.98,1.01) | 0.702 | 0.99 (0.98,1.00) | 0.078 | 0.99 (0.98,1.00) | 0.001 |
| Maternal smoking-pregnancy | 1.17 (0.82,1.68) | 0.388 | 0.89 (0.30,2.60) | 0.828 | 1.61 (0.56,4.64) | 0.374 | 1.01 (0.34,2.99) | 0.990 | 1.59 (1.05,2.39) | 0.027 |
| Parental asthma during pregnancy | 1.09 (0.80,1.49) | 0.591 | 2.25 (1.09,4.67) | 0.029 | 0.84 (0.27,2.56) | 0.753 | 2.65 (1.23,5.71) | **0.013** | 1.72 (1.20,2.45) | 0.003 |
| Maternal hay-fever pregnancy | 0.79 (0.58,1.09) | 0.149 | 1.07 (0.48,2.38) | 0.869 | 1.31 (0.52,3.33) | 0.565 | 1.04 (0.43,2.48) | 0.937 | 1.28 (0.90,1.82) | 0.176 |
| Maternal eczema during pregnancy | 0.98 (0.67,1.44) | 0.932 | 1.70 (0.71,4.06) | 0.234 | 0.76 (0.19,3.07) | 0.702 | 2.19 (0.91,5.27) | 0.079 | 1.39 (0.90,2.14) | 0.138 |
| Breast feeding | 1.19 (0.84,1.66) | 0.325 | 0.74 (0.33,1.68) | 0.473 | 1.35 (0.41,4.42) | 0.617 | 1.34 (0.47,3.82) | 0.579 | 0.96 (0.64,1.45) | 0.858 |

**Table S14:** Early life and parental risk factors associated with trajectories in second replication analysis; NIFW-NL as a reference. Data are relative risk ratios (Wald 95% CI) from the replication cohorts and Wald χ^2^ p values. For all continuous variables, the relative risk ratios are per unit increase, whereas for categorical variables, the relative risk ratios are for “yes”, with “no” as a reference. RRR: relative risk ratio. Empty cells represent cells with no or limited cases.

|  | **ETW NLF** | | **NIFW RLF** | | **LOW NLF** | | **PEW NLF** | | **PEW RLF** | |
| --- | --- | --- | --- | --- | --- | --- | --- | --- | --- | --- |
| **Risk Factors** | **RRR (95% CI)** | **P-value** | **RRR (95% CI)** | **P-value** | **RRR (95% CI)** | **P-value** | **RRR (95% CI)** | **P-value** | **RRR (95% CI)** | **P-value** |
| Male | 1.5 (1.04, 2.16) | 0.03 | 1.65 (1.27, 2.15) | <0.001 | 0.92 (0.69, 1.23) | 0.57 | 1.6 (1.12, 2.28) | 0.01 | 1.64 (0.89, 3.00) | 0.11 |
| Prematurity | 1.83 (0.91, 3.70) | 0.09 | 1.79 (1.06, 3.03) | 0.03 | 0.79 (0.37, 1.69) | 0.55 | 2.23 (1.18, 4.21) | 0.01 | 2.77 (1.05, 7.32) | 0.04 |
| Birth weight in kg | 0.78 (0.56, 1.10) | 0.16 | 0.65 (0.52, 0.83) | <0.001 | 0.97 (0.74, 1.28) | 0.85 | 0.63 (0.46, 0.88) | 0.01 | 1.46 (0.81, 2.64) | 0.21 |
| Maternal smoking-pregnancy | 1.48 (0.88, 2.50) | 0.14 | 1.13 (0.75, 1.70) | 0.57 | 0.88 (0.54, 1.42) | 0.60 | 1.52 (0.92, 2.50) | 0.10 | 2.19 (1.03, 4.66) | 0.04 |
| Partner smoking-pregnancy | 0.97 (0.59, 1.61) | 0.92 | 0.97 (0.67, 1.39) | 0.86 | 1.03 (0.70, 1.51) | 0.90 | 1.43 (0.92, 2.22) | 0.11 | 0.95 (0.42, 2.16) | 0.90 |
| Maternal history of asthma | 2.95 (1.82, 4.80) | <0.001 | 2.08 (1.40, 3.09) | <0.001 | 2.7 (1.81, 4.02) | <0.001 | 3.35 (2.12, 5.29) | <0.001 | 3.13 (1.46, 6.71) | <0.001 |
| Paternal history of asthma | 2.21 (1.30, 3.78) | <0.001 | 1.83 (1.20, 2.79) | <0.001 | 1.47 (0.91, 2.38) | 0.12 | 2.86 (1.76, 4.65) | <0.001 | 4.76 (2.37, 9.58) | <0.001 |
| Deliver type-Normal | 0.96 (0.54, 1.69) | 0.88 | 0.79 (0.54, 1.16) | 0.23 | 1.12 (0.70, 1.79) | 0.63 | 0.85 (0.50, 1.44) | 0.55 | 0.8 (0.33, 1.94) | 0.63 |
| Ever breastfed by 15 months | 0.63 (0.14, 2.85) | 0.55 | 0.44 (0.16, 1.19) | 0.11 | 1.12 (0.25, 5.03) | 0.88 | 1.42 (0.18, 10.98) | 0.74 |  |  |

**REFERENCES:**

1. Boyd A, Golding J, Macleod J, Lawlor DA, Fraser A, Henderson J, Molloy L, Ness A, Ring S, Davey Smith G. Cohort Profile: The 'Children of the 90s'--the index offspring of the Avon Longitudinal Study of Parents and Children. *Int J Epidemiol* 2013; **42**(1): 111-27.

2. Fraser A, Macdonald-Wallis C, Tilling K, et al. Cohort Profile: the Avon Longitudinal Study of Parents and Children: ALSPAC mothers cohort. *Int J Epidemiol* 2013; **42**(1): 97-110.

3. Northstone K, Lewcock M, Groom A, et al. The Avon Longitudinal Study of Parents and Children (ALSPAC): an update on the enrolled sample of index children in 2019. *Wellcome Open Res* 2019; **4**: 51.

4. Harris PA, Taylor R, Thielke R, Payne J, Gonzalez N, Conde JG. Research electronic data capture (REDCap)--a metadata-driven methodology and workflow process for providing translational research informatics support. *J Biomed Inform* 2009; **42**(2): 377-81.

5. Custovic A, Simpson BM, Murray CS, et al. The National Asthma Campaign Manchester Asthma and Allergy Study. *Pediatr Allergy Immunol* 2002; **13**(s15): 32-7.

6. Kurukulaaratchy RJ, Fenn M, Twiselton R, Matthews S, Arshad SH. The prevalence of asthma and wheezing illnesses amongst 10-year-old schoolchildren. *Respir Med* 2002; **96**(3): 163-9.

7. Kurukulaaratchy RJ, Fenn MH, Waterhouse LM, Matthews SM, Holgate ST, Arshad SH. Characterization of wheezing phenotypes in the first 10 years of life. *Clin Exp Allergy* 2003; **33**(5): 573-8.

8. Arshad SH, Holloway JW, Karmaus W, et al. Cohort Profile: The Isle Of Wight Whole Population Birth Cohort (IOWBC). *Int J Epidemiol* 2018; **47**(4): 1043-4i.

9. Wang G, Hallberg J, Bergstrom PU, et al. Assessment of chronic bronchitis and risk factors in young adults: results from BAMSE. *Eur Respir J* 2021; **57**(3).

10. Miller MR, Hankinson J, Brusasco V, et al. Standardisation of spirometry. *Eur Respir J* 2005; **26**(2): 319-38.

11. Beydon N, Davis SD, Lombardi E, et al. An official American Thoracic Society/European Respiratory Society statement: pulmonary function testing in preschool children. *Am J Respir Crit Care Med* 2007; **175**(12): 1304-45.

12. Elliott L, Henderson J, Northstone K, Chiu GY, Dunson D, London SJ. Prospective study of breast-feeding in relation to wheeze, atopy, and bronchial hyperresponsiveness in the Avon Longitudinal Study of Parents and Children (ALSPAC). *J Allergy Clin Immunol* 2008; **122**(1): 49-54, e1-3.

13. Cucco A, Simpson A, Haider S, et al. Patterns of Respiratory Symptoms and Asthma Diagnosis in School-Age Children: Three Birth Cohorts. *Allergy* 2025; **80**(7): 1923-34.

14. Kurukulaaratchy RJ, Raza A, Scott M, et al. Characterisation of asthma that develops during adolescence; findings from the Isle of Wight Birth Cohort. *Respir Med* 2012; **106**(3): 329-37.

15. Roberts G, Peckitt C, Northstone K, et al. Relationship between aeroallergen and food allergen sensitization in childhood. *Clin Exp Allergy* 2005; **35**(7): 933-40.

16. Reddel HK, Taylor DR, Bateman ED, et al. An official American Thoracic Society/European Respiratory Society statement: asthma control and exacerbations: standardizing endpoints for clinical asthma trials and clinical practice. *Am J Respir Crit Care Med* 2009; **180**(1): 59-99.

17. Deliu M, Fontanella S, Haider S, et al. Longitudinal trajectories of severe wheeze exacerbations from infancy to school age and their association with early‐life risk factors and late asthma outcomes. *Clinical & Experimental Allergy* 2020; **50**(3): 315-24.

18. Quanjer PH, Stanojevic S, Cole TJ, et al. Multi-ethnic reference values for spirometry for the 3-95-yr age range: the global lung function 2012 equations. *Eur Respir J* 2012; **40**(6): 1324-43.

19. Granell R, Henderson AJ, Sterne JA. Associations of wheezing phenotypes with late asthma outcomes in the Avon Longitudinal Study of Parents and Children: A population-based birth cohort. *J Allergy Clin Immunol* 2016; **138**(4): 1060-70 e11.

20. Russell AE, Ford T, Russell G. The relationship between financial difficulty and childhood symptoms of attention deficit/hyperactivity disorder: a UK longitudinal cohort study. *Soc Psych Psych Epid* 2018; **53**(1): 33-44.

21. Nawa N, Black MM, Araya R, Richiardi L, Surkan PJ. Pre- and post-natal maternal anxiety and early childhood weight gain. *J Affect Disorders* 2019; **257**: 136-42.

22. Matvienko-Sikar K, K ON, Fraser A, et al. Maternal prenatal anxiety and depression and trajectories of cardiometabolic risk factors across childhood and adolescence: a prospective cohort study. *Bmj Open* 2021; **11**(12): e051681.

23. Nagin DS, Jones BL, Passos VL, Tremblay RE. Group-based multi-trajectory modeling. *Stat Methods Med Res* 2018; **27**(7): 2015-23.

24. Klijn SL, Weijenberg MP, Lemmens P, van den Brandt PA, Passos VL. Introducing the fit-criteria assessment plot - A visualisation tool to assist class enumeration in group-based trajectory modelling. *Statistical Methods in Medical Research* 2017; **26**(5): 2424-36.

25. Niyonkuru C, Wagner AK, Ozawa H, Amin K, Goyal A, Fabio A. Group-Based Trajectory Analysis Applications for Prognostic Biomarker Model Development in Severe TBI: A Practical Example. *J Neurotraum* 2013; **30**(11): 938-45.

26. McHugh ML. Interrater reliability: the kappa statistic. *Biochem Medica* 2012; **22**(3): 276-82.

27. Rand WM. Objective Criteria for the Evaluation of Clustering Methods. *Journal of the American Statistical Association* 1971; **66**(336): 846-50.
